# RF-photonic deep learning processor with Shannon-limited data movement

**DOI:** 10.1126/sciadv.adt3558

**Published:** 2025-06-11

**Authors:** Ronald Davis, Zaijun Chen, Ryan Hamerly, Dirk Englund

**Affiliations:** ^1^ Research Laboratory of Electronics, MIT, Cambridge, MA 02139, USA.; ^2^ Ming Hsieh Department of Electrical and Computer Engineering, University of Southern California, Los Angeles, CA 90089, USA.; ^3^ NTT Research Inc., PHI Laboratories, 940 Stewart Drive, Sunnyvale, CA 94085, USA.

## Abstract

Edholm’s law predicts exponential growth in data rate and spectrum bandwidth for communications. Owing to exponentially increasing deep neural network computing demands and the slowing of Moore’s law, new computing paradigms are required for future advanced communications like 6G. Optical neural networks (ONNs) are promising accelerators but struggle with scalability and system overhead. Here, we introduce our multiplicative analog frequency transform optical neural network (MAFT-ONN), an artificial intelligence hardware accelerator that experimentally computes fully analog deep learning on raw radio frequency (RF) signals, performing modulation classification that quickly converges to 95% accuracy. MAFT-ONN also exhibits scalability with nearly 4 million fully analog operations for MNIST digit classification. Because of the Shannon capacity–limited analog data movement, MAFT-ONN is also hundreds of times faster than traditional RF receivers.

## Teaser

MAFT-ONN is a photonic AI hardware accelerator that performs fully analog deep learning on raw RF signals.

## INTRODUCTION

Artificial intelligence (AI) has been revolutionizing a broad range of fields, including radio frequency (RF) signal processing and advanced communications. In environments where the spectrum is congested with several users and many channels, hand-engineered systems are becoming increasingly infeasible. Here, AI can be leveraged to process the increasingly complex spectral environment while meeting the rising demand for higher wireless data rates. Signal processing AI enables the next generation of communications such as 6G, where capabilities like cognitive radio, fingerprinting, and dynamic resource allocation may take pivotal roles (*1*–*3*).

For RF signal processing, state-of-the-art AI approaches first digitize the IQ data and then either convert the signal into an *N* × 2 IQ tensor (*4*, *5*) or compute the spectrogram (*6*, *7*) (or other time-frequency transform) to convert the signal into an image. The preprocessed signal is then inserted into a convolutional neural network (CNN) or other deep learning model for tasks like signal classification or device fingerprinting.

However, while digital processors can compute CNNs with high accuracy, these methods introduce substantial latency that make real-time spectrum processing impossible for digital processors. This is because state-of-the-art digital architectures require several steps to move the large volume of RF data to and from the compute. The alternative is to store the data for later offline analysis, but this is not feasible for time-sensitive tasks. In addition, these current approaches struggle to maintain high performance while keeping cost, size, weight, and power low due to the need to use high-performance devices like FPGAs, GPUs, and RFSoCs (*8*–*10*).

Optical systems promise AI acceleration by encoding, routing, and processing analog signals in optical fields, allowing for operation at the quantum noise limit with high bandwidth and low energy consumption. Optical neural network (ONN) schemes rely on (i) performing linear algebra intrinsically in the physics of optical components and/or (ii) in-line nonlinear transformations. For (i), past approaches include Mach-Zehnder interferometer meshes (*11*–*15*), on-chip micro-ring resonators (*16*–*19*), wavelength-division multiplexing (*20*–*22*), photoelectric multiplication (*23*), spatial light modulation (*24*–*29*), optical scattering (*30*), optical attenuation (*31*), vertical-cavity surface-emitting lasers (*32*), and optical diffraction (*33*–*36*). For (ii), past approaches include optical-electrical-optical elements (*14*, *31*, *37*–*39*) and all-optical (*18*, *28*, *40*–*43*) approaches. However, to fully take advantage of the potential ultralow latency and energy consumption available in photonics, it is necessary to implement linear and nonlinear operations together with minimal overhead. Simultaneously achieving (i) and (ii) in a way that preserves high hardware scalability and performance has been an open challenge.

**Fig. 1.**
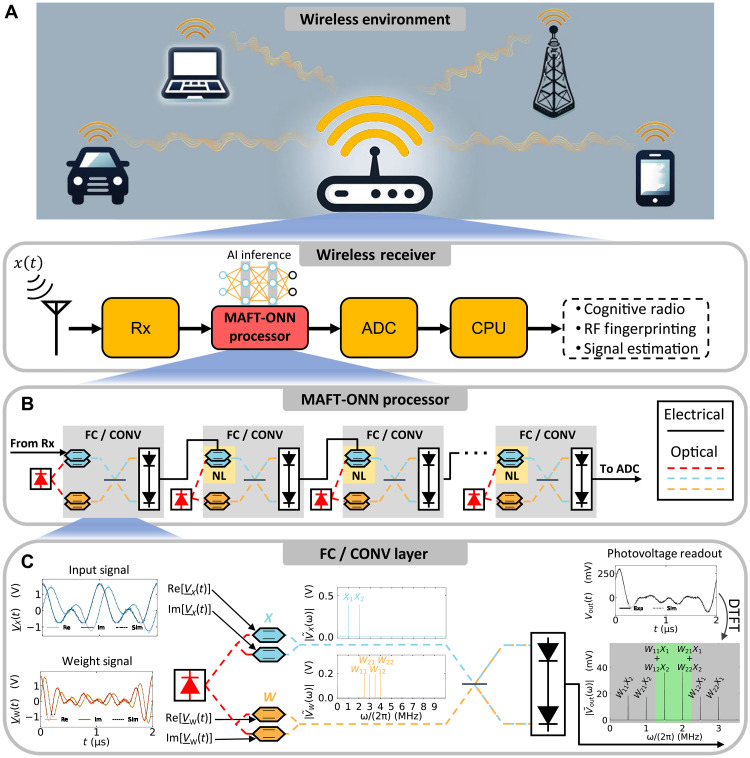
An overview of the MAFT-ONN architecture. (**A**) The MAFT-ONN processor accelerates both traditional signal processing operations and AI inference for waveforms like radio waves. The analog received waveform is fed into MAFT-ONN for fully analog processing, after which the output may be read out digitally using an analog-to-digital converter (ADC) or fed into another analog system. (**B**) An outline of the MAFT-ONN architecture. Each photoelectric multiplication physically computes either a fully connected (FC) or a 1D convolution (CONV) layer. The nonlinear activation (NL) for each layer physically corresponds to the nonlinear region of the following modulator. The units can be cascaded to implement several DNN layers fully in analog with no digital overhead. (**C**) A close-up of a single FC or CONV layer. For an FC layer, the weight signal is programmed such that the photoelectric multiplication yields a matrix-vector product in the frequency domain, where the green region in the frequency domain is isolated with a filter. For a CONV layer, all frequencies of the output signal are used. S. Wilcox (MIT) aided in designing the figure.

Our multiplicative analog frequency transform optical neural network (MAFT-ONN) architecture simultaneously achieves (i) and (ii) for deep neural network (DNN) inference with high scalability in both DNN size and layer depth. We experimentally demonstrate the MAFT-ONN in a three-layer DNN for inference of Modified National Institute of Standards and Technology (MNIST) images and modulation classification. In this architecture, we encode neuron values in the amplitude and phase of frequency modes, and “photoelectric multiplication” (*23*) performs matrix-vector products in a single shot. The nonlinear activation for each layer is achieved using the nonlinear region of an electro-optic modulator, thus enabling a scalable front-to-back photonic hardware accelerator for DNNs. Figure 1A contextualizes use cases for the MAFT-ONN processor.

## MAFT-ONN ARCHITECTURE

As illustrated in Fig. 1B, a series of DNN layers corresponds to a cascading of photoelectric multiplications where each one computes either a fully connected (FC) or 1D convolution (CONV) layer. The nonlinear activation (NL) for all neurons in a given layer is achieved by operating in the nonlinear regime of the following modulator.

Figure 1C details an experimental example of a 2 × 2 matrix-vector product using the MAFT scheme. In practice, the electrical voltage signal $V_{X}\left(t\right)$ is the incoming RF waveform and $V_{W}\left(t\right)$ is the weight signal generated by MAFT-ONN. With an underbar denoting the complex representation of the signal, the terms $\text{Re}\left[\underline{V}\left(t\right)\right]$ and $\text{Im}\left[\underline{V}\left(t\right)\right]$ represent a voltage signal and its $90^{\circ }$ phase-shifted copy, the combination of which is required to achieve single-sideband suppressed carrier (SSB-SC) modulation. (See Methods for experimental details.)

The SSB-SC modulated input and weight signals are then photoelectrically multiplied to yield the output voltage signal: $V_{\text{out}}\left(t\right)\propto \text{Im}\left[{\underline{V}}_{X}^{*}\left(t\right){\underline{V}}_{W}\left(t\right)\right]$ . The output signal’s Fourier Transform ${\tilde{V}}_{\text{out}}\left(\omega \right)$ corresponds to the desired matrix multiplication performed in a single shot. This matrix multiplication is achieved by appropriately programming the frequency content of the weight signal $V_{W}\left(t\right)$ . For an FC layer, the frequencies of ${\tilde{V}}_{\text{out}}\left(\omega \right)$ that correspond to the matrix product are within the green region in Fig. 1C and are isolated using a bandpass filter. For a CONV layer, all frequencies remain. See section SG for the generalized matrix multiplication algorithm.

## RESULTS

### MNIST digit inference

**Fig. 2.**
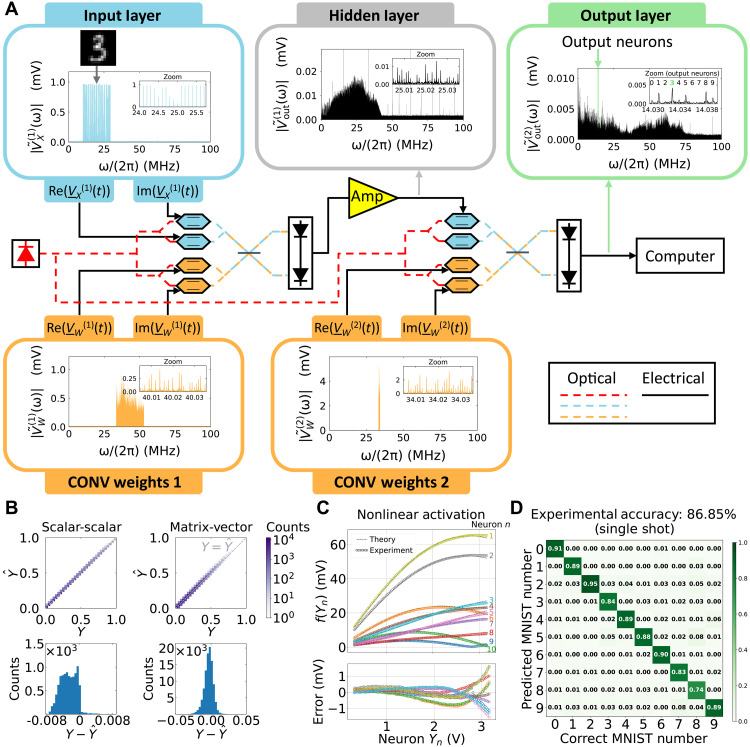
Experimental demonstration of MAFT-ONN. (**A**) An example of an experimental inference of a 14 × 14 MNIST image encoded into the input signal $V_{X}^{\left(1\right)}\left(t\right)$ . The two convolutions are achieved with the weight signals $V_{W}^{\left(1\right)}\left(t\right)$ and $V_{W}^{\left(2\right)}\left(t\right)$ , respectively. The nonlinear activation is achieved using an amplifier so that the signal $V_{\text{out}}^{\left(1\right)}\left(t\right)$ reaches the nonlinear region of the next DPMZM. As shown in the zoom plot of $V_{\text{out}}^{\left(2\right)}\left(t\right)$ , the image is correctly classified. (**B**) In the upper half, 2D histograms compare the experimental output values $\hat{Y}$ to the expected curve fitted value Y . In the lower half, 1D histograms plot the error $Y-\hat{Y}$ . The scalar-scalar plot contains 10,000 randomized 1 × 1 matrix products, yielding 9-bit precision compared to the curve fit. The matrix-vector plot contains 10,000 randomized 10 × 10 matrix products (thus 100,000 values), yielding 8-bit precision. (**C**) An experimental characterization of the nonlinear activation function of an MZM. We programmed $V_{X}^{\left(1\right)}\left(t\right)$ as a 10 × 1 input vector, and gradually increased its amplitude until it reached the nonlinear regime of the MZM. We then curve fitted an analytical model to the experimental data. (**D**) A confusion matrix of the experimental three-layer DNN over 10,000 14 × 14 MNIST images, yielding an experimental accuracy of 86.85%.

We experimentally demonstrated a proof of concept of the MAFT-ONN architecture for a three-layer DNN trained to classify MNIST digits. As shown in Fig. 2A, the DNN consists of two CONV weight signals with a nonlinear activation for the hidden layer. The frequency-encoded input layer consists of a flattened 14 × 14 MNIST image and thus contained 196 frequencies that represent the neurons. This was convolved with a weight CONV kernel of 19,600 frequencies to yield the hidden layer of 39,100 neurons (frequencies). After using a dual-parallel Mach-Zehnder modulator (DPMZM) as the nonlinear activation, the hidden layer was next convolved with a second weight CONV kernel of 1000 frequencies to yield the output layer of 10 neurons, one output neuron for each of the MNIST digits.

The number of multiply-and-accumulates (MACs) computed per MNIST image inference is the sum of the MACs in two CONV layers. The number of MACs computed for a 1D convolution between two vectors with lengths $N_{1}$ and $N_{2}$ is simply $N_{1}N_{2}$ . This is because the number of MACs when the two vectors fully overlap (assuming $N_{2}>N_{1}$ ) is $\left(N_{2}-N_{1}+1\right)N_{1}$ and the number of MACs when the convolution is at the edges is $2\sum_{i=1}^{N_{1}-1}i=\left(N_{1}-1\right)N_{1}$ . Therefore, the number of MACs experimentally computed per MNIST inference is: (14·14)·19,600+1000·10=3,851,600 MACs. The first term is from convolving the flattened input image with the weights and the second term only counts the MACs used for the output neurons.

The three-layer experimental DNN inferred 10,000 14 × 14 MNIST images, where the digital DNN has an accuracy of 92.52% and the experimental DNN has an accuracy of 86.85%. One contribution to the experimental inaccuracy is ripples found in the experimental nonlinear activation function, perhaps due to the path length difference of the interferometer (see section SC for data on this). Other methods to increase the accuracy include introducing voltage drivers to the DPMZMs to increase the SNR by an order of magnitude (see Methods) and performing the DNN training in situ on the hardware itself (*44*). The confusion matrix of the experimental DNN is shown in Fig. 2D.

### LTI signal processing

The MAFT architecture is capable of computing arbitrary finite impulse response (FIR) linear time invariant (LTI) operations in the frequency domain. When interpreting the frequency modes as discrete LTI samples, the weight signal $V_{W}\left(t\right)$ can be programmed to implement arbitrary frequency-domain FIR LTI convolutions with the input signal $V_{X}\left(t\right)$.

**Fig. 3.**
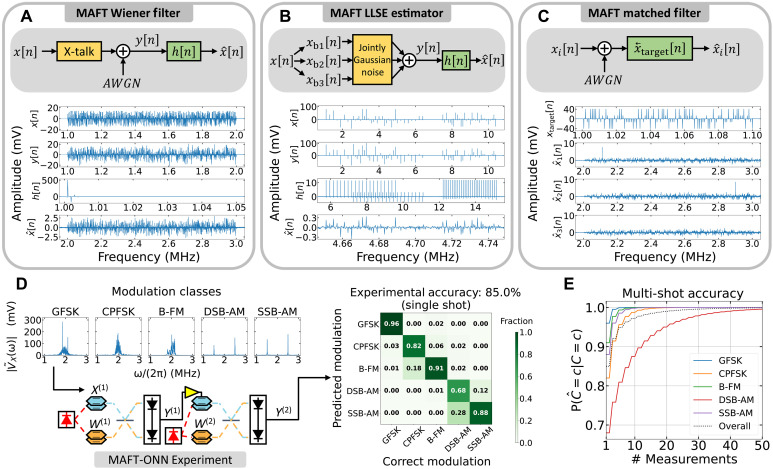
Experimental demonstrations of the signal processing capabilities of MAFT-ONN. The signal processing operations in (A) to (C) used the experimental setup in Fig. 1C. (**A**) The frequency-domain LTI framework was used to implement a Wiener filter that recovers a signal that suffered from frequency–cross-talk and AWGN. (**B**) The matrix-vector product method from Fig. 1C was used to implement an LLSE estimator to recover a signal that suffered jointly Gaussian noise in various frequency bands. (**C**) The frequency-domain LTI framework was used to scan the spectrum of a signal for a specific frequency signature. (**D**) The three-layer DNN hardware in Fig. 2A was used to experimentally implement modulation classification on raw RF signals using MAFT-ONN. For single-shot inference, the modulation classification experimentally achieved 85.0% accuracy compared to the digital accuracy of 89.2%. (**E**) With the experimental single-shot accuracy as the baseline, we show that a majority vote multimeasurement scheme will asymptotically improve the accuracy to 100%. With only five measurements, the accuracy improves to 95%. B-FM, broadcast frequency modulation; CPFSK, continuous phase frequency shift keying; DSB-AM, double sideband amplitude modulation; GFSK, Gaussian frequency shift keying; SSB-AM, single sideband amplitude modulation.

Let the input voltage signal be $V_{X}\left(t\right)=\sum_{n=1}^{N}X_{n}\text{cos}\left(n\cdot \Delta \omega \cdot t\right)$ and the LTI filter signal be $V_{W}\left(t\right)=\sum_{r=1}^{R}W_{r}\text{cos}\left(r\cdot \Delta \omega \cdot t\right)$ . Then, the frequency-domain LTI interpretation is: $x\left[n\right]\equiv X_{n}\to n\Delta \omega$ and $w\left[n\right]\equiv W_{n}\to n\Delta \omega$ . Hence, the values of x[n] and w[n] are mapped to the frequency mode at nΔω . With this interpretation, appropriately programming the SSB-SC conditions of the DPMZMs enables MAFT-ONN to compute convolutions that correspond to $y\left[n\right]=\left(x^{*}*w\right)\left[n\right]=\sum_{k=-\infty }^{\infty }x^{*}\left[k\right]w\left[n-k\right]$ . Therefore, MAFT yields an LTI convolution for real-valued signals and a still useful LTI-like operation for complex-valued signals. See section SN for the mathematical derivation of the LTI framework from physics principles. Figure 3 (A to C) illustrates experimental results of various signal processing operations.

#### FIR Wiener filter

Let $V_{X}\left(t\right)$ be a frequency channelized signal with 1000 frequency modes where the amplitude of each frequency encodes a symbol. Here, we assume a 4-bit encoding scheme where each symbol is equiprobable a priori. After $V_{X}\left(t\right)$ is generated, cross-talk is introduced between each of the frequency modes and then additive white Gaussian noise (AWGN) is added to yield the distorted signal $V_{Y}\left(t\right)$ . The goal is to estimate the original signal $V_{X}\left(t\right)$ from the received signal $V_{Y}\left(t\right)$.

The frequency content of $V_{X}\left(t\right)$ and $V_{Y}\left(t\right)$ is modeled as jointly wide-sense stationary (WSS) random processes x[n] and y[n] , respectively. Given that the frequency cross-talk and AWGN are known, we calculate the covariance matrices to construct an FIR Wiener filter h[n] of length 50, meaning that the previous 50 samples of y[n] will be used to estimate the next sample. The estimated signal $\hat{x}\left[n\right]$ is then computed by applying the Wiener filter to yield: $\hat{x}\left[n\right]=\left(y*h\right)\left[n\right]$.

The effectiveness of the Wiener filter is evaluated using the mean squared error (MSE) between the original signal and the estimated signal. The MSE is calculated over 10 experimental measurements of the instance of the random process. The experimental MSE of the uncorrected signal ${|\;x-y\;|}^{2}$ has a mean of 39.46 ${\text{mV}}^{2}$ and an SD of 0.27${\text{mV}}^{2}$ , and the experimental MSE of the corrected signal ${|\;x-\hat{x}\;|}^{2}$ has a mean of 24.59 ${\text{mV}}^{2}$ and an SD of 0.14 ${\text{mV}}^{2}$ , yielding an average MSE improvement of 37.69%. This closely matches the theoretical MSE improvement of 38.78%. Figure 3A plots one of the measurements for each signal.

According to Parseval’s theorem, estimating $V_{X}\left(t\right)$ in the frequency domain (opposed to the time domain) is effective because the energy of the error signal is the same in both the time and frequency domains. Hence, given the appropriate context, the MAFT frequency-domain estimation is a practical tool for signal processing scenarios.

#### LLSE estimator

The linear least squares estimator (LLSE) is more general than the Wiener filter and works on non-WSS random processes. Let $V_{X}\left(t\right)$ be frequency channelized as in the previous example, except this time it is split up into three frequency bands each with different amplitude encoding characteristics. In frequency-domain LTI notation, $V_{X}\left(t\right)$ is broken into its three frequency bands as $x\left[n\right]=x_{b1}\left[n\right]+x_{b2}\left[n\right]+x_{b3}\left[n\right]$.

Each of the three bands goes through a combination of shared and individual sources of noise, which is modeled as jointly Gaussian noise, to yield the distorted signal y[n] . Because each of the frequency bands have different encoding schemes and different sources of noise, x[n] and y[n] are no longer WSS and cannot be estimated using a Wiener filter.

Assuming we have knowledge of the jointly Gaussian noise, we constructed the optimal LLSE filter by calculating the appropriate covariance matrices. We use the scheme to create the filter h[n] that implements the LLSE estimator to produce $\hat{x}\left[n\right]$.

As before, 10 experiments of the instance were measured to calculate the experimental MSE. The experimental uncorrected MSE ${|\;x-y\;|}^{2}$ has a mean of 479.58 ${\text{mV}}^{2}$ with an SD of 1.03 ${\text{mV}}^{2}$ , and the experimental corrected MSE ${|x-\hat{x}|}^{2}$ has a mean of 193.38${\text{mV}}^{2}$ and an SD of 22.98 ${\text{mV}}^{2}\;$for an average MSE improvement of 59.68%. This, again, is close to the theoretical MSE improvement of 63.17%. Figure 3B plots one of the measurements for each signal.

#### Matched filters

A useful benefit of MAFT is that a single photoelectric multiplication can be used to simultaneously scan the entire RF spectrum for a target signal. Let $V_{i}\left(t\right)$ be a series of signals that we wish to scan for a specific frequency signature ${\tilde{V}}_{\text{target}}\left(\omega \right)$ . The frequency-domain LTI representations of $V_{i}\left(t\right)$ and ${\tilde{V}}_{\text{target}}\left(\omega \right)$ are $x_{i}\left[n\right]$ and $x_{\text{target}}\left[n\right]$ , respectively.

Figure 3C shows three examples of applying the matched filter to various received signals. For $x_{1}\left[n\right]$ , the target $x_{\text{target}}\left[n\right]$ is located lower in the spectrum, and thus, a peak appears there in the matched filter output. For $x_{2}\left[n\right],$ the target is located higher in the spectrum so the peak appears there. The signal $x_{3}\left[n\right]$ is a control that does not contain the target.

### Modulation classification

Modulation classification entails identifying various schemes used to wirelessly transmit information, used in scenarios like cognitive radio. Using the same experimental setup in Fig. 2A, the input activation signal $V_{X}^{\left(1\right)}\left(t\right)$ consists of a frame from a synthetically generated waveform across five different types of modulation: Gaussian frequency shift keying, continuous phase frequency shift keying, broadcast frequency modulation, double sideband amplitude modulation, and single sideband amplitude modulation as illustrated in Fig. 3D. The channel model includes fading due to Rician multipath, phase and frequency offsets due to clock offsets, timing drifts due to clock offsets, and AWGN such that the SNR is 30. This three-layer DNN experimentally achieved 85.0% single-shot modulation classification accuracy over 500 input activation frames compared to the digital single-shot accuracy of 89.2%.

Figure 3E illustrates the performance of a straightforward scheme to use multiple consecutive measurements to boost the accuracy using the single-shot experimental accuracy as the baseline. When MAFT-ONN makes multiple inferences, the final inference can be determined with a “majority vote” scheme, where the final inference is simply the class with the most inferences. The diagonal elements of the confusion matrix in Fig. 3D yield $\mathbb{P}\left(\hat{C}=c|C=c\right)$ , which is the conditional probability that the inference $\hat{C}$ will be correct given the input class C . The majority vote scheme assumes (i) that each measurement is statistically independent, (ii) the experimental single-shot accuracy represents the conditional probability for all incoming samples, (iii) the modulation class does not change in the duration of measurements, and (iv) only those five classes arrive at MAFT-ONN. With this scheme, the conditional probability of correct detection quickly approaches 100%, while reaching 95% with only 5 measurements.

We note that the chosen modulation classes are inherently more oriented toward frequency-domain encoding compared to modulation schemes like quadrature amplitude modulation, binary phase shift keying (BPSK), and quadrature phase shift keying (QPSK). When analyzing phase-modulated signals like BPSK in the frequency domain, the phase-encoded information is highly condensed around the carrier frequency. An input signal with information highly condensed in the frequency domain is problematic for MAFT-ONN due to the loss of computational density. As visualized in Fig. 1C, for a given input signal, increasing the frequency resolution of the weight kernel elements will increase the density of computations. Thus if the input signal is already too condensed in the frequency domain, creating weight signals with even denser frequency modes will incur an impractical amount of readout time.

This problem is alleviated by spreading the phase-encoded information into the frequency domain, enabling enough computational density to achieve the desired operation. As a proof of concept, we trained a digital twin of MAFT-ONN to distinguish between BPSK and QPSK with 90% accuracy with a single convolutional layer. In the digital twin, the phase information was spread into the frequency domain by appending an analog preprocessor before MAFT-ONN that applied an envelope detector and separately extracted the instantaneous frequency.

## DISCUSSION

To our knowledge, the MAFT-ONN architecture is the first hardware accelerator that performs AI inference on raw communications signals without digitization or preprocessing. Our several experimental demonstrations show that MAFT-ONN is flexible enough to implement a powerful combination of fully analog frequency-domain LTI processing and AI inference to enable new high-performance signal processing capabilities with the benefits of the low power consumption, cost, size, and weight of optical systems. The power consumption of optical systems with different architectures but similar components has already been extensively studied (*14*, *23*); thus, section SO analyzes the communication link gain of MAFT-ONN.

### Computational throughput

The throughput T measures the number of multiply-and-accumulates (MACs) computed within a given time.

$$T=\frac{\#\text{MACs}}{\text{latency}}$$

The number of MACs computed in an FC layer with N input neurons and R output neurons is N·R MACs. A CONV layer can be interpreted as keeping the spurious frequencies in an FC layer; thus, the number of MACs in a CONV layer with N input neurons and N·R weight neurons is $N^{2}\cdot R$ MACs. The time it takes to read out the output signal is the Gabor limit, which is $\frac{1}{\text{min}\left(\Delta f,f_{0}\right)}$ , where Δf is the smallest frequency spacing of the output signal and $f_{0}$ is the lowest neuron frequency of the output signal. (Note that 2πf=ω , where ω is the angular frequency from the previous sections.)

Section SI derives the theoretical throughput, showing that the throughput for an FC layer asymptotically approaches the available bandwidth B for modulating signals: $T_{\text{FC}}\approx B$ , and similarly for the throughput of a CONV layer: $T_{\text{CONV}}\approx N\cdot B$.

Hence, for the MNIST classification experiment in Fig. 2A, the achieved throughput is $T=\frac{3,851,600\;\text{MACs}}{120\;\text{ns}+1\;\text{ms}}=3.85$ giga-operations/s, where the 120 ns is the time-of-flight latency and the 1 ms is the readout latency due to the 1-kHz frequency spacing. The throughput of our experimental setup is limited by a bandwidth of 43 MHz, because the amplifier supports a minimum bandwidth of 2 MHz and the preceding balanced photodetector supports a maximum bandwidth of 45 MHz (see Methods for further component details). Given that the second balanced photodetector has a bandwidth of 150 MHz, the theoretical maximum throughput for the two CONV layers is 196·43MHz+10·150MHz≈10 giga-operations/second. The theoretical throughput can be achieved by exploiting the anti-aliasing characteristics near DC (section SI) and using wider frequency spacing (section SD).

For future scaling of MAFT-ONN, the bandwidth B limiting the throughput is not the RF bandwidth of the electrical components but the available optical bandwidth, for example, the 20 THz among the S, C, and L telecommunications bands. See section SM for an analysis of scalable MAFT-ONN architectures that take advantage of the full optical bandwidth and multiplexing.

Therefore, the combination of using the full optical bandwidth (on the order of terahertz) and spatial multiplexing (on the order of a hundred) (*45*) immediately yields a straightforward path to reaching peta-operation per second scale throughputs using MAFT-ONN with current technology. Thus, the MAFT-ONN is competitive with electronic counterparts like the Google TPUv3 that has a throughput greater than 400 tera-operations per second (*46*).

The throughput for a given bandwidth B is doubled by using complex-valued operations. The encoding scheme for computing complex-valued matrix products with MAFT-ONN is derived in section SL.

### System-level latency

**Fig. 4.**
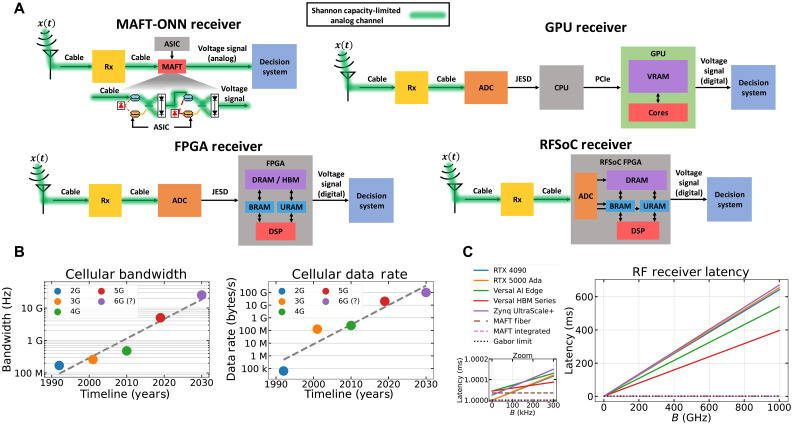
System-level latency analysis comparing the MAFT-ONN architecture to state-of-the-art digital architectures. (**A**) Modeling various high-performance computing architectures in the context of receiving an RF signal x(t) , implementing a filter on it, and then sending it to a “decision system” that performs some operation based on the inferred qualities of the processed x(t) . The green highlights show that the analog information “flows” into and through MAFT-ONN, and thus the amount of information moving through the MAFT-ONN processor is limited by the Shannon capacity of the analog channels feeding it information (in the absence of nonideal factors like multipath fading and interference). (**B**) An example of Edholm’s law applied to mobile communications. This shows that wireless mobile bandwidth and data rates have been and will likely continue to exponentially grow. (**C**) Using the models in (A), the latency for processing x(t) with increasing bandwidth B is estimated for various high-performance processors. We assume that the digital processors are performing at peak specifications with 100% processor and memory utilization in ideal conditions. For simplicity, we assume that all FPGA components are running at 500 MHz. Because MAFT-ONN avoids the bottleneck of digital data movement, its overall latency improves by two orders of magnitude compared to traditional receivers.

Figure 4A illustrates how state-of-the-art digital architectures require several steps to move data to and from the compute (where the compute elements are the red subblocks). This bottleneck is only being accentuated by Edholm’s law, which predicts that the amount of bandwidth and data rates for communications will continue to exponentially rise. Figure 4B illustrates an example of Edholm’s law for mobile communications, plotting the year each mobile network generation was introduced and its maximum bandwidth and data rate. We compare the theoretical system-level latency for various RF receiver architectures in Fig. 4C, where system-level latency is defined as the duration of time between the signal first arriving at the antenna to the time the decision-making system can extract the desired information from the output voltage signal.

In this scenario, we assume that the incoming signal x(t) has bandwidth B and that the computations for x(t) require frequency resolution Δf . For simplicity, we assume that the preselector circuit labeled “Rx” contributes negligible latency and that the ADC speed exactly matches the required Nyquist sampling rate for B . Together, B and Δf determine the number of points $N=2\left\lceil \frac{B}{\Delta f}\right\rceil$ required to digitally capture x(t) . Therefore, as B increases, the amount of digital data that must be moved to/from memory also increases, thereby raising the digital latency.

We used the models in Fig. 4A to estimate the theoretical latencies for the digital processors in Fig. 4C. We assumed that all processors and data lanes are operating: at their theoretical peak speeds, with 100% processor and memory utilization, with no processing or memory management overhead, with no data rate degradation from high clock speeds, with no throttling due to overheating, and limitless DRAM/HBM in which to store RF data. In practice, these processors may experience more than an order of magnitude slower latency than their theoretical optimal performance. The FGPAs will likely incur less overhead than the GPUs because the FGPAs trade off having less management overhead by storing less memory.

Per the scenario in Fig. 4A, after x(t) is processed into an output voltage signal, it reaches a “decision system” that performs the appropriate operation based on the output signal’s qualities. For the traditional receiver architectures, the output voltage signal is digital (this contribution to the digital latency is neglected). For MAFT, the output voltage signal is an analog waveform, so the decision system extracts the desired information from the waveform to perform its operation. MAFT’s decision system may or may not use an ADC, for example, a non-ADC decision system may consist of a bank of bandpass filters followed by an analog power selector circuit. Regardless of whether an ADC is used, the latency of MAFT’s decision system is fundamentally dominated by the Gabor limit.

As the zoom subplot in Fig. 4C illustrates, some digital processors theoretically have superior latency at low bandwidths, but even then are quickly overtaken by MAFT as the bandwidth increases. At 15 GHz, MAFT’s latency ranges from 7 to 11 times faster than digital architectures. As the input signal bandwidth increases, so does MAFT’s latency benefit. For example, when considering scenarios like 5G multiple-input multiple-output where there are massive volumes of RF data streaming into the receiver, the combined instantaneous bandwidth is much larger than that of a single antenna receiver. Figure 4C illustrates that at 1000 GHz, MAFT’s latency improvements range from 400 to 670 times faster than digital architectures, assuming the MAFT-ONN multiplexing architectures in section SM are used to reach 1000 GHz of bandwidth. The latency benefit from MAFT is physically attributed to the fast data movement to the compute. The green highlighted paths in Fig. 4A illustrate that the analog data “flows” into and through MAFT.

As demonstrated in the experiments portrayed in Fig. 3, the ability for MAFT to perform AI or linear processing on RF signals is agnostic to the type of modulation or coding scheme used for the input signal. Thus, the amount of data that can simultaneously be processed by MAFT is limited by the capacity of the wireless link feeding the input signal, given that the MAFT components can support that capacity. For the former, the capacity of a wireless link in a realistic scenario depends on several factors including attenuation from weather, multipath from structures, and wireless congestion. As shown in Fig. 4B, the peak data rate for which 5G is designed is 20 Gbps. Theoretically, given the 5-GHz bandwidth from Fig. 4B and a strong SNR of 25 dB, the theoretical Shannon capacity for a 5G link is ~40 Gbps. Studies show that 6G may yield 5 to 10 times (or higher) improvements over 5G data rate capacities (*47*, *48*).

Therefore, for MAFT to process 5G and 6G signals, the optical components must achieve at least those capacities. While the theoretical Shannon capacity of such optical components is currently an unsolved problem (*49*, *50*), there exist approximations such as the nonlinear Shannon limit (*50*–*52*). The theoretical capacity of MAFT is best estimated by considering intensity-modulated direct detection (IMDD) links, which have been extensively studied and use the same optical components as MAFT. Data rates ranging from 100 to 200 Gbps per optical wavelength have been experimentally demonstrated for IMDD links (*53*–*55*), showing that the optical components in the MAFT architecture fundamentally support such capacities. Hence, especially with the multiplexing schemes detailed in section SM, MAFT is comfortably poised to support 5G and 6G links at their capacities.

The time-of-flight latency per photoelectric multiplication of the MAFT-ONN experiment was measured to be 60 ns per layer, which matches the expected latency given the propagation through the DPMZM, fiber, and photodetector. The readout latency was 1 ms, determined by the Gabor limit. See section SP for a breakdown of our latency estimation models. Here, MAFT-ONN promises orders of magnitude faster latency compared to high-performance digital architectures while benefiting from the superior size, weight, power, and cost of photonic circuits. Because MAFT-ONN uses conventional components found in nanophotonic foundries, fabrication into a photonic integrated circuit is straightforward.

We have introduced and demonstrated our MAFT analog computing scheme and MAFT-ONN hardware accelerator for scalable, fully analog DNN acceleration that uses frequency-encoded neurons for convolutional and FC layers. The MAFT-ONN architecture yields much flexibility for running various types and sizes of DNNs without changing the hardware.

This architecture is also the first AI hardware accelerator that directly performs inference on raw RF signals without digitization or preprocessing, offering faster latency than FPGAs while simultaneously benefiting from the cost, size, weight, and power consumption of optics. The combination of frequency-domain LTI signal processing and trainable weight signals enables a flexible, high-performance computing paradigm. In addition, when using the full optical bandwidth and spatial multiplexing, the throughput of this processor is also competitive with other state-of-the-art AI hardware accelerators. Future work includes increasing the scale of MAFT-ONN using wavelength-division and spatial multiplexing.

## METHODS

### 2 × 2 matrix multiplication experiment

For the example experiment in Fig. 1C that details a 2 × 2 matrix multiplication, $V_{X}\left(t\right)$ and $V_{W}\left(t\right)$ were generated by an arbitrary waveform generator (AWG) and then sent to DPMZMs without using voltage drivers. These DPMZMs perform SSB-SC modulation of the signals, without which the modulated signals would be dual-sideband and cancel each other out after the photoelectric multiplication.

To SSB-SC modulate a signal using a DPMZM, one copy of the signal is sent to one of the arms of the DPMZM while a $90^{\circ }$ phase-shifted copy is sent to the other arm. Thus, let an underbar indicate an analytical Hilbert transform, yielding ${\underline{V}}_{X}\left(t\right)=H_{a}\left[V_{X}\left(t\right)\right]$ . Then, $\text{Re}\left[{\underline{V}}_{X}\left(t\right)\right]=V_{X}\left(t\right)$ is the original signal, and $\text{Im}\left[{\underline{V}}_{X}\left(t\right)\right]$ is the $90^{\circ }$ phase-shifted copy. Although the 90° phase shift was generated using an AWG in this experiment, in practice, this phase shift can be achieved using commercial passive RF phase shifters.

### Three-layer MNIST DNN

We experimentally demonstrated the three-layer DNNs using the MAFT-ONN scheme with the apparatus shown in Fig. 2A. An AWG generates $V_{X}^{\left(1\right)}\left(t\right)$ , $V_{W}^{\left(1\right)}\left(t\right)$ , and $V_{W}^{\left(2\right)}\left(t\right)$ , all of which are modulated into the optical domain using DPMZMs directly from the AWG without using voltage drivers, as the 1.5-V peak output from the AWG was sufficient. Note that the accuracy can be improved by introducing drivers to increase the SNR by an order of magnitude because the 1.5-V peak AWG output corresponds to +16.5 dBm of power, whereas each DPMZM can safely handle a maximum power of +28 dBm. The photoelectric multiplication of $V_{X}^{\left(1\right)}\left(t\right)$ and $V_{W}^{\left(1\right)}\left(t\right)$ yielded $V_{\text{out}}^{\left(1\right)}\left(t\right)$ after the first layer. We then amplified $V_{\text{out}}^{\left(1\right)}\left(t\right)$ to reach the nonlinear regime of the DPMZM in the second layer, after which it became the input signal to the hidden layer. For convenience, we only used one submodulator of the DPMZM for this signal, thus modulating $V_{\text{out}}^{\left(1\right)}\left(t\right)$ in the dual-sideband suppressed carrier (DSB-SC) mode. For the hidden layer, we programmed $V_{W}^{\left(2\right)}\left(t\right)$ as the next CONV kernel. The multiplication of the DSB-SC–modulated $V_{\text{out}}^{\left(1\right)}\left(t\right)$ and the SSB-SC–modulated $V_{W}^{\left(2\right)}\left(t\right)$ results in a copy of $V_{\text{out}}^{\left(2\right)}\left(t\right)$ appearing further up in the spectrum, as can be seen in the plot of $Y^{\left(2\right)}$ in Fig. 2A. Last, the analog output of the second layer $V_{\text{out}}^{\left(2\right)}\left(t\right)$ was sampled digitally and Fourier transformed. See section SA for more details on the three-layer DNN experimental setup.

The input activations are downsampled 14 × 14 MNIST images that are represented by the frequency-encoded signal $V_{X}^{\left(1\right)}\left(t\right)$ containing 196 frequencies spaced at 100 kHz. The input activation is convolved by a weight kernel $V_{W}^{\left(1\right)}\left(t\right)$ containing 19,600 frequencies spaced at 1 kHz to yield the signal of the hidden layer, $V_{\text{out}}^{\left(1\right)}\left(t\right)$ . Note that $V_{W}^{\left(1\right)}\left(t\right)$ was programmed as for an FC layer, but we chose to keep the “spurious” frequencies, thus effectively making it a CONV operation. Next, the hidden layer $V_{\text{out}}^{\left(1\right)}\left(t\right)$ , is multiplied by the second layer weight signal $V_{W}^{\left(2\right)}\left(t\right)$ that contains 1000 frequencies spaced at 1 kHz to yield the output signal $V_{\text{out}}^{\left(2\right)}\left(t\right)$.

The signal $V_{W}^{\left(1\right)}\left(t\right)$ was designed so that the frequency content of $V_{\text{out}}^{\left(1\right)}\left(t\right)$ would maximize use of the 43 MHz of bandwidth of the components. Thus, the frequency content of $V_{\text{out}}^{\left(1\right)}\left(t\right)$ is spread between 2 MHz (amplifier limit) and 45 MHz (photodetector limit). The frequency spacing of 1 kHz was used for $V_{W}^{\left(1\right)}\left(t\right)$ and $V_{W}^{\left(2\right)}\left(t\right)$ to maximize the number of MACs computed within that 43 MHz (thus increasing the expressivity of the DNN) while balancing out a reasonable readout time.

Physically, the minimum frequency spacing achievable is limited by the linewidth of the RF frequency modes, which may be widened from phase noise. Equipment-wise, it is limited by the RAM of the AWG for creating long time-series signals or the RAM of the data acquisition device. Application-wise, it is limited by the required readout speed.

We implemented the one-hot vector that represents the output MNIST values by randomly selecting a set of 10 adjacent frequencies among the frequencies of $V_{\text{out}}^{\left(2\right)}\left(t\right)$ to demonstrate the flexibility of our scheme. (See section SK for a performance analysis of using sparse FC layers for DNN training.) The 10 output neuron frequencies were randomly chosen to be 14.03 to 14.039 MHz, with 1 kHz spacing. The zoom of the plot of $V_{\text{out}}^{\left(2\right)}\left(t\right)$ in Fig. 2A shows the mapping of the neuron frequencies to the MNIST digits, where the digit is classified by the frequency mode with that largest magnitude. Hence, the final readout only considers the absolute value of the frequencies modes, making the one-hot vector insensitive to phase.

For the MNIST classification, we programmed real-valued positive and negative neuron values into both the input vectors and weight matrices. Negative neuron values are physically represented by a π phase shift in that particular frequency mode, allowing for analog matrix algebra with negative numbers.

An analytic model of the hardware was used to train the DNN offline, similar to previous ONN training schemes that account for the transfer function of the hardware (*33*, *56*, *57*). The offline training produced a set of weight matrices that were then encoded into the RF signals used for the experimental inferences. See sections SB, SC, and SE for details on the offline DNN characterization and training.

### Three-layer modulation classification DNN

The same hardware setup used for the MNIST classification in Fig. 2A was also used to implement the three-layer DNN for modulation classification, so we reuse the mathematical notation. The input activation waveforms were generated using MATLAB (*58*). Each waveform had a random time delay applied to model receiving the signals at unknown times. The power of all the frames was normalized at baseband and then upconverted to 1 MHz as shown in Fig. 3C.

The first DNN layer was CONV with the DPMZM biases configured such that $V_{W}^{\left(1\right)}\left(t\right)$ functioned as a trainable LTI filter. Following the first CONV layer was the DPMZM nonlinearity. Then, as portrayed in Fig. 2A, the second-layer weight signal $V_{W}^{\left(2\right)}\left(t\right)$ was DSB-SC modulated and implemented another CONV layer. The LTI weight signal $V_{W}^{\left(1\right)}\left(t\right)$ consisted of 50 frequency modes spaced at ~9 kHz and the second-layer weight signal $V_{W}^{\left(2\right)}\left(t\right)$ consisted of 6143 frequency modes spaced at ~200 Hz. Both $V_{W}^{\left(1\right)}\left(t\right)$ and $V_{W}^{\left(2\right)}\left(t\right)$ were modeled as complex signals, so the number of trainable parameters was double the number of frequency modes (one for magnitude and phase).

For the one-hot vector readout of $V_{\text{out}}^{\left(2\right)}\left(t\right)$ , instead of choosing adjacent frequencies like in the MNIST classification, we chose frequencies that were evenly spread out across the spectrum as we found that this allowed the weights to train most effectively, and as with our MNIST inference, the absolute values of the frequencies were measured so that the frequency mode with the largest magnitude was considered the DNN inference.

## Supplementary Materials

### This PDF file includes:

Supplementary Text Figs. S1 to S14 Tables S1 to S7 References
