# Supplementary material for: RF-photonic deep learning processor with Shannon-limited data movement
Source: Sci Adv. 2025 Jun 11;11(24):eadt3558. doi: 10.1126/sciadv.adt3558 (PMC12153973; doi:10.1126/sciadv.adt3558)
Supplement: Supplementary file 1 — Supplementary Text Figs. S1 to S14 Tables S1 to S7 References [file sciadv.adt3558_sm.pdf]

Science Advances
AAAS

Supplementary Materials for

RF-photonic deep learning processor with Shannon-limited data movement

Ronald Davis III *et al.*

Corresponding author: Ronald Davis III, radavis4@mit.edu

Sci. Adv. **11**, eadt3558 (2025)
DOI: 10.1126/sciadv.adt3558

This PDF file includes:

Supplementary Text
Figs. S1 to S14
Tables S1 to S7
References

EXPERIMENT

A. 3-Layer DNN Hardware

The experimental setup for the 3-layer DNN inference is shown in the main text. All of the optics is done in-fiber using commercial components. A total of four DP-MZMs are used for the 3-layer experiment. As conveyed in the main text, two pairs of DPMZMs implement the two CONV photoelectric multiplications.

The first set of DPMZMs have ~ 25 GHz bandwidth (Exail MXIQER-LN-30), where all the fibers both before and after the modulator are polarization-maintaining (PM) with FC/APC leads. The second set of DPMZMs have ~ 15 GHz bandwidth (Thorlabs LN865-FC), where all the fibers before the modulator are PM, but all the fibers after are single-mode (SM) with FC/PC leads. Thus, we demonstrate that this architecture works with both types of fiber. The balanced photodetectors (Thorlabs PDB450C) had adjustable gains. For the MNIST experiment, the first was set to 10^4 V/A gain with 45 MHz bandwidth, and the second set to 10^3 V/A gain with 150 MHz bandwidth. For the modulation classification experiment, both were set to the 10^4 V/A gain.

As explained in the main text, the nonlinear activation was achieved by using an amplifier to push the signal into the nonlinear domain of the next DPMZM. For the MNIST experiment, we used an amplifier (MPA-11-40) with 40 dB gain that operates from 2 MHz - 1 GHz. And thus we observe the bandwidth limitation for the MNIST experiment—the frequency content of the hidden layer must lie between 2 MHz (the amplifier) and 45 MHz (the balanced photodetector). Thus, the frequencies of the weight kernel were programmed to maximize use of the bandwidth while staying within the bounds of the hardware constrains. For the modulation classification experiment, we used an amplifier (Exail DR-AN-20-HO) with 27 dB gain that operates from 80 kHz - 27 GHz.

Since every DPMZM has 3 DC biases (one for each sub-MZM and then a phase bias for interfering the sub-MZMs together), each of the DPMZMs was controlled using Exail DPMZM bias controllers, which biased all the DPMZMs for SSB-SC modulation. The bias controller does this by biasing each of the sub-MZMs at their minimum point, and then biasing the phase. Note that the minimum intensity bias point of an MZM is usually highly nonlinear when using a single photodetector. This is because for an electric field $E(t)$, the output of the signal photodetector is proportional to $|E(t)|^2$, the intensity. This means that for a bias ϕ_b, the single photodetector output will yield a bias curve of $\sin(\phi_b)^2 = \frac{1}{2}(1 - \cos(2\phi_b))$. However, the output of a balanced photodetector is proportional to not the intensity, but the electric field $|E(t)|$, yielding a bias curve of $\sin(\phi_b)$. Thus, for a value of $\phi_b = 0$, we see that this is the minimum bias point of the intensity, but is the positive quadrature bias point of the electric field. This conveniently allows us to compute linear matrix operations while operating in the SSB-SC mode.

After setting the bias points for all the modulators, the bias would remain stable for > 24 hours without active control. The Thorlabs DPMZMs require DC-blocks at the RF inputs to stabilize the bias points.

Figure S1 shows how the interferometers are stabilized. The entire experiment is on an optical table that does not have air stabilization. Each interferometer is placed on an optical breadboard that is lifted by vibration-absorbing feet. The surface of each optical breadboard is also covered with vibration-abosrbing material on which the fibers and components are placed. The fibers are also taped down to minimize movement. Both interferometers are boxed to reduce temperature and air current fluctuations.

We use one 1550nm external cavity single-frequency laser (Thorlabs SFL1550P) with a temperature and power controller (Thorlabs CLD1015). The laser is split into four paths using three 1×2 fiber splitters; a laser path for each of the four DPMZMs. Although the laser is pig-tailed with a PM fiber at the output, we observed the best stability when using fiber polarizers after the laser and before the DPMZMs.

The frequency-encoded RF signals were generated using two Keysight M3202A AWGs that output ± 1.5V peak signals. Given that the $V\pi$ for the DPMZMs ranged from $4.5V$ to $6V$, no voltage drivers were used between the AWG and DPMZMs. Each DPMZM requires two RF inputs to achieve SSB-SC modulation; one copy of the RF signal at one sub-modulator, and other copy of the RF signal with a 90° phase shift at the other modulator. Thus, two AWG channels were used for each DPMZM. Since one of the DPMZMs is driven by the output of the first layer, we used 6 AWG channels total for the 3-layer inference experiment (not including using one channel to ground the unused sub-MZM on the DPMZM operating in the DSB-SC mode).

For accurate results, the triggers of the AWG signals must be synchronized. This is because any time delay will cause a gradient in the phases between all the frequencies, thus changing the behavior of the photoelectric multiplication. As explained in the main text, we measured a 60 ns time-of-flight latency per layer, so we programmed the weight signal for the second layer weight matrix with a corresponding time delay for MNIST inference. For the modulation classification we trained on signals with randomized time delays anyways so we did not adjust the second layer weight signal timing.

We used a spectrum analyzer (BK Precision 2682) to read the amplitude of the output frequencies for the MNIST inference and an oscilloscope (Agilent DSO81004A) plus taking the absolute value of the Fast-Fourier Transform (FFT) for the modulation classification.

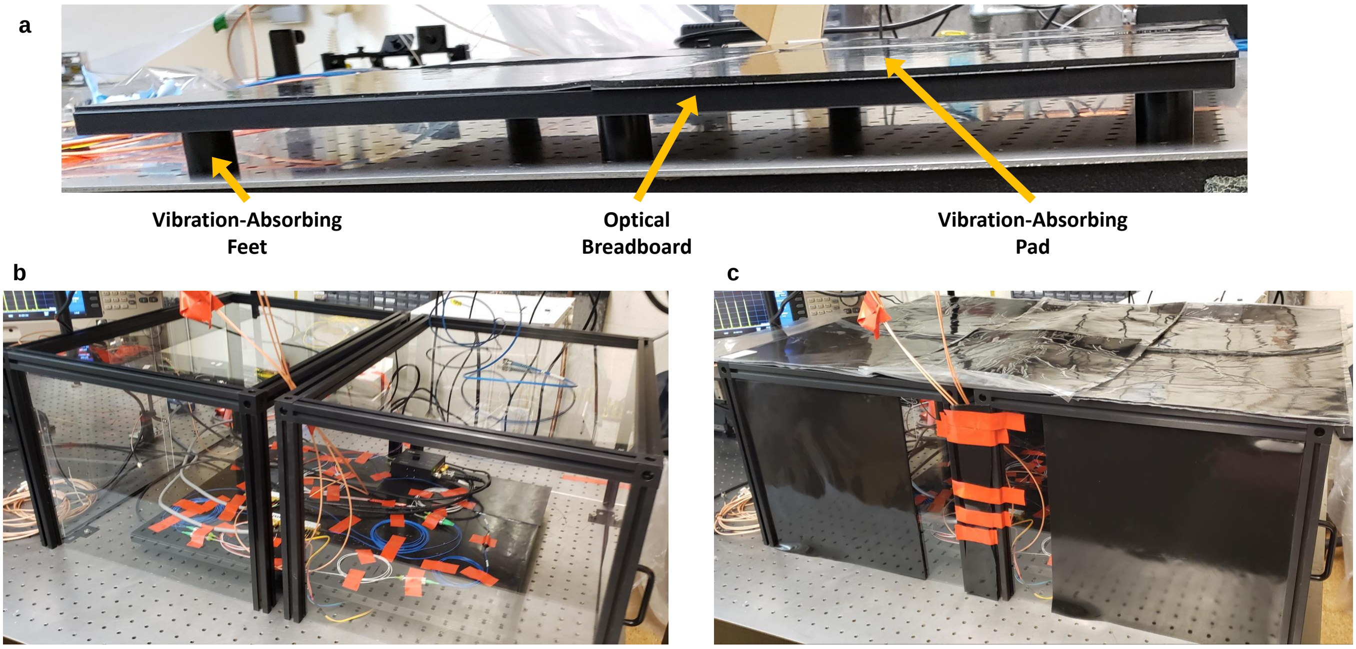

Figure S1. The stabilization of the fiber interferometer that implements the first photoelectric multiplication in the 3-layer DNN experiment. The whole setup is on a single optical table that does not have air stabilization. (a) Each interferometer has its own optical breadboard, supported by vibration-absorbing pads. (b) Each interferometer was boxed to reduce temperature and air current fluctuations. (c) More padding was placed on the box to reduce vibrations.

B. Linear Curve Fit Characterizations

Note that the following mathematical analysis uses the notation from Figure 2A in the main text. All references to figures in the main text will be explicitly stated. All the linear curve fit experiments use real values that are programmed to be positive or negative using a π phase shift.

Mathematical Analysis: To test linear matrix-vector multiplication, we measure the photovoltage response $V_{\text{out}}^{(1)}(t)$ using a spectrum analyzer that scans the relevant part of the bandwidth to extract $V_Y^{(1)}(t)$. Here, the input laser is modulated by $V_X^{(1)}(t)$ and $V_W^{(1)}(t)$ via DPMZMs in the linear regime. We repeat this multiplication over randomized values of $X^{(1)}$ and $W^{(1)}$ to obtain the full set of characterization data.

To measure the accuracy of the matrix products, we use a theoretical model to compare with the experiment. The result of linearly modulating the input vector is:

$$\begin{aligned} f(V_X^{(1)}(t)) &= \chi_0 + \chi_1 e^{i\omega_{\text{LD}} t} \cdot \text{H}_a \left[\sin\left(\chi_2 V_X^{(1)}(t) + \chi_3 \right) \right] \\ &\approx \chi_1 \chi_2 e^{i\omega_{\text{LD}} t} \cdot \text{H}_a \left[V_X^{(1)}(t) \right] \\ &\approx \chi_1 \chi_2 E_X^{(j)}(t) \end{aligned}$$

where χ_0 contributes to the DC offset; χ_1 depends on the laser power, insertion loss, and propagation loss; χ_2 depends on the V_π and efficiency of the MZM; and χ_3 depends on the bias conditions and inherent bias point of the MZM. We assume that $\chi_0 = \chi_3 = 0$ in the linear regime. Similarly, the linear modulation of the weight matrix yields $f(V_W^{(1)}(t)) \approx \chi_1 \chi_2 E_W^{(1)}(t)$. Therefore the resulting photoelectric multiplication is:

$$\begin{aligned} V_{\text{out}}^{(1)}(t) &= \chi_{PD} \text{Im} \left[\left(\chi_1 \chi_2 E_X^{(j)}(t) \right)^* \chi_1 \chi_2 E_W^{(j)}(t) \right] \\ &= \chi_{PD} \left(\chi_1 \chi_2 \right)^2 \text{Im} \left[\left(E_X^{(j)}(t) \right)^* E_W^{(j)}(t) \right], \end{aligned}$$

where χ_{PD} is determined by the responsivity of the photodetector and the termination resistance.

Hence for the linear characterization, we use a 1-parameter curve fit where the parameter estimates the value of $\chi_{PD} \left(\chi_1 \chi_2 \right)^2$. To attain the curve fit parameter, we used a single randomized matrix-vector product and gradually increased the amplitude to create a curve, where the slope of the curve is determined by $\chi_{PD} \left(\chi_1 \chi_2 \right)^2$. We re-calibrated the curve fit whenever we changed the size of the matrix-vector product being experimentally computed. See Supplementary Section B for more details on the statistical linear curve fitting methods.

The main text Figure 2B shows the experimental matrix-vector multiplication performance of our architecture, where Y is the expected curved-fitted value of the output vector, and $\hat{Y}$ is the experimental output vec-

tor. Both Y and $\hat{Y}$ are normalized to the largest value among all the products. First, we characterized scalar-scalar products by computing 10,000 randomized scalar-scalar multiplications and comparing them to the curve fitted analytical product, yielding 9-bit precision. Next, we computed 10,000 randomized 10×10 matrix-vector products to yield 8-bit precision. Thus, we achieved accurate experimental linear matrix-vector products using this architecture.

Experimental Methods: Most experimental measurements of the neuron frequency modes throughout both the main text and supplementary used a spectrum analyzer (SA), excluding the measurements in the main text Figure 1C, Figure 2A, Figure 2D, or other supplementary figures with a time-domain measurement that used an oscilloscope. Since SAs can only discern the magnitude of each frequency mode and not the phase, all SA measurements took the absolute value of the actual neuron value. This does not prevent the experiment from computing negative-valued matrix algebra.

All the linear measurements with randomized values that determined a bit precision (main text Figure 2B and supplementary Figure S2(b)) used a single readout from the SA (no averaging). Additionally, the linear curve fits in Figures S3(b) and S3(c) also used a single readout per experimental data point. The frequency correction measurements in S3(d) used the averaging function of the SA to average 100 samples, as that was a one-time deterministic characterization.

All curve fits were computed using the function “curve_fit” from “scipy.optimize” in Scipy version 1.6.2, which provided both the curve fit parameters and the standard deviation errors. Additionally, all curve fits are “double-sided,” allowing for both positive and negative deviations.

AWG Measurements

For the linear curve fit, we set a baseline by characterizing the bit-precision of the AWG. Figure S2(a) shows the experimental setup, where the AWG is directly connected to the SA. This experiment consisted of generating single tone 20 MHz sine waves with known amplitudes X, measuring the amplitudes on the SA with a marker set on 20 MHz to yield $\hat{X}$, then normalizing X and $\hat{X}$ to the largest value, and finally comparing X with $\hat{X}$. Note that SAs excel at relative frequency measurements, but require calibration for absolute measurements. Thus, we first ran the measurement with 100 randomized values, then deterministically found the calibration factor by adjusting the linear scaling until the bit precision was maximized. The SA calibration factor was found to be ~ 0.68. We then ran the measurement again with this factor for 1,000 randomized values, where the results are shown in Figure S2(b). We measured a normalized standard deviation of $2.8 \cdot 10^{-4}$ to yield 12-bit precision.

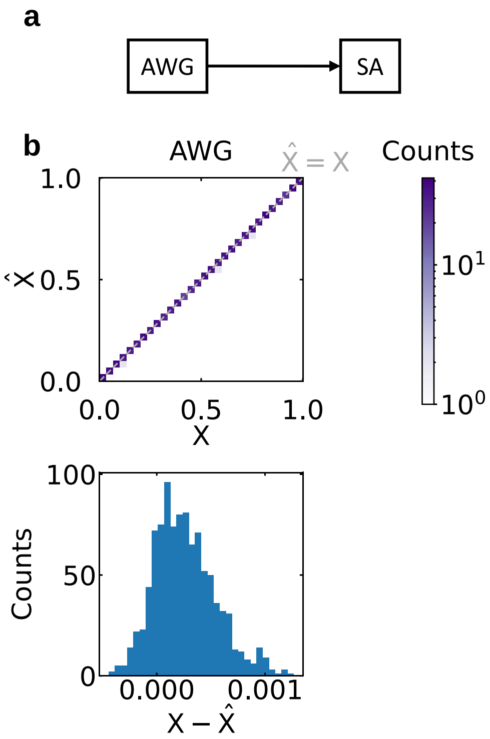

Figure S2. Characterizing the bit-precision of the AWG. (a) The output of the AWG is directly measured by an SA. (b) A comparison between the expected value X and the measured value $\hat{X}$ for 1,000 random scalars, where each scalar is simply the amplitude of a single frequency mode. Above is a 2D histogram comparing the values of X and $\hat{X}$, and below is a 1D histogram of the error $X - \hat{X}$. This measurement yielded 12-bit precision for the AWG.

Scalar-Scalar Product Measurements

Figure S3(a) shows the experimental setup used for the scalar-scalar and matrix-vector characterizations in the main text. We estimated the value of $\chi_{PD} (\chi_1 \chi_2)^2$ using a 1-parameter curve fit for the linear characterizations. We absorb the aforementioned SA calibration factor into all the curve fits.

The scalar-scalar product can be interpreted as a 1×1 matrix-vector multiplication, and thus is set up in the same way as the matrix-vector characterization. We used a single randomized scalar-scalar product to determine the curve fit. The input vector X was randomly set to a 20 MHz sinusoidal wave with peak amplitude of 0.8140314 V. The weight matrix W was randomly set to -0.88271151 V on a 2 MHz sinusoidal wave. Thus the output Y was a 18 MHz sinusoidal wave. Since the AWG has a setting to scale the amplitudes of the signals, we gradually increased the amplitude of X to create the curve in Figure S3(b). The standard deviation error of this curve fit is $8.87 \cdot 10^{-5}$, where the units of the output signal is in volts.

Thus, we accurately computed 10,000 randomized products using a single curve fit from a random scalar-scalar product. This measurement yielded a normalized standard deviation of $2.01 \cdot 10^{-3}$, corresponding to 9-bit precision. We note that the errors the scalar-scalar error in Figure 2C are primarily negative with a normalized mean of $-2.75 \cdot 10^{-3}$. This is mostly likely due to the curve fit method where we used a single randomized scalar-scalar product to predict the behavior of the following 10,000 products, demonstrating efficient device characterization. The error of the single randomized product used to characterize the device happened to be above the average value of the following products.

Matrix-Vector Product Measurements

The experimental setup in Figure S3(a) was also used for the matrix-vector product measurements. In this case, the input vector X was a 10-frequency signal from 11 MHz to 20 MHz spaced at 1 MHz. The weight signal W was a 100-frequency signal from 30.6 MHz to 40.5 MHz spaced at 100 kHz.

For this measurement we included a deterministic correction for the uneven frequency response of the system. After testing different photodectors and AWGs, we found that it was the DPMZMs that have a significantly varying frequency response even within kHz bandwidth. We individually characterized each DPMZM in the setup illustrated in Figure S3(a) by using the AWG to send a flat RF frequency comb at the frequencies of interest to the DPMZM under test, and then sending a 100 MHz single tone signal to the other DPMZM. This yields an output where the RF frequency comb is simply shifted by 100 MHz, but the shape of the output RF frequency comb reveals the frequency response of the DPMZM under test.

Figure S3(d) shows the result of these measurements. The orange stems are the experimentally measured relative amplitudes of the frequency modes, characterizing the frequency response of the DPMZM. The blue stems are all set to a normalized amplitude of exactly 1 to contrast the orange stems. To apply this correction to the experiment, we multiply X and W by these frequency corrections before generating their signals from the AWG.

With these frequency corrections, we then used a randomized 10×10 matrix-vector product for the curve fit in Figure S3(c). This 1-parameter curve fit yielded a standard deviation error of $2.71 \cdot 10^{-7}$, where the output signal was in units of volts. At this point there is one last deterministic calibration required, which is to account for the scaling of the AWG. That is, we must scale the peak amplitude of every signal to 1 V before sending it through the AWG (which then has its own amplitude setting). Although this scaling factor is different for every signal, it is deterministic and known beforehand. Thus, we multiply the curve fit parameter found from Figure S3(c) by this scaling factor to find the true parameter of the system.

Then we compute 10,000 randomized 10×10 matrix-vector products, where we apply the AWG scaling to each matrix-vector product. This leads to a normalized standard deviation of $6.43 \cdot 10^{-3}$, corresponding to the 8-bit precision presented in the main text. The normalized mean is $-3.41 \cdot 10^{-3}$.

C. Nonlinear Curve Fit Characterizations

Note that the following mathematical analysis uses the notation from Figure 2A in the main text. All references to equations and figures to the main text will be explicitly stated. All data contains real-valued positive and negative numbers.

Mathematical Analysis: The main text Figure 2C illustrates a nonlinear curve fit for a simple intensity-modulated direct detection (IMDD) link, which consists of an electrical input signal $V_X^{(1)}(t)$ being modulated by an MZM and then immediately detected with a photodetector. Our equation that models the output of the IMDD link is:

$$V_{\text{out}}(t) = \chi_0 + \chi_{PD}\chi_1 \sin\left(\chi_2 V_X^{(1)}(t) + \chi_3\right).$$

Thus, we use a 4-parameter curve fit for the nonlinear characterization (counting $\chi_{PD}\chi_1$ as a single parameter). The main text Figure 2C shows an example of curve fitting the analytical model to an experimental characterization of an MZM, where $V_X^{(1)}(t)$ is a 10×1 input vector.

Experimental Methods: We also characterized the nonlinear behavior of modulators from various manufacturers and configurations to show the accuracy and applicability of our physics-based DNN model. All our nonlinearity characterizations are 4-parameter curve fits to the equation:

$$f(V_X(t)) = \chi_0 + \chi_1 \sin\left(\chi_2 V_X(t) + \chi_3\right),$$

where $V_X(t)$ is the input vector signal. Note that we absorb all physical effects into the four parameters above, including χ_{PD}, the SA calibration factor, etc. And like the linear characterizations, all the nonlinear curve fits are also "double-sided."

Figure S4 shows several configurations for which we curve fitted the nonlinearity. These measurements were performed using the same method as the linear curve fit; by using the AWG amplitude setting to gradually increase the amplitude of the input vector X until it reached the nonlinear regime of the modulator under test. Each configuration within Figure S4 has a different randomized input vector. The DNN algorithm in Supplementary Section E was adjusted to match each curve fit configuration. All configurations except the one in Figure S4(e) used the frequency correction method described in Supplementary Section B. An RF amplifier was used

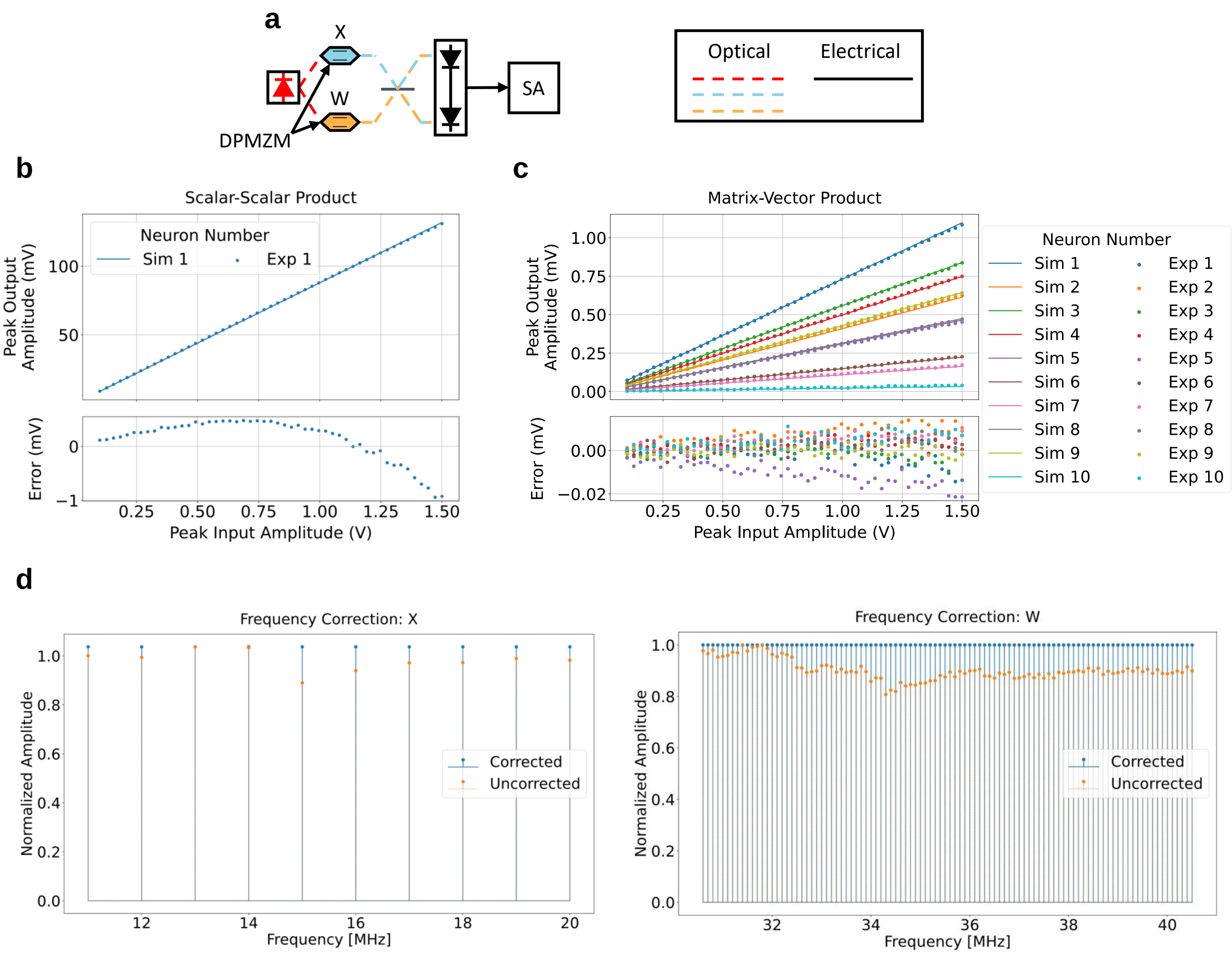

Figure S3. The linear matrix-vector characterizations. (a) The experimental setup for the linear characterization. (b) The curve fit used for the scalar-scalar characterization in the main text. (c) The curve fit used for the matrix-vector characterization in the main text. (d) The deterministic corrections to account for the uneven frequency response of the DPMZMs for the matrix-vector characterization in (c).

for all configurations to increase the power of the electrical signal to reach the nonlinear regime of the modulator under test. Table S1 shows the standard deviation error of the various nonlinear curve fits. All measurements in Figures S4(a)-(d) used the averaging function of the SA to average 50 samples for each experimental data point. The data points in the 3-layer DNN characterization measurement in Figure S4(e) did not use the SA averaging feature, instead using a single SA readout per experimental data point.

Figure S4(a) is a simple IMDD link, using a regular MZM biased at quadrature (not a DPMZM). All other configurations exclusively use DPMZMs. This MZM has a polarization-maintaining (PM) fiber lead input and a single-mode (SM) fiber lead output. Thus, all optical fibers before the MZM are PM, and the ones after are SM.

**Figure**	χ_0 Error	χ_1 Error	χ_2 Error	χ_3 Error
4(a)	$4.52\cdot10^{-4}$	$1.05\cdot10^{-5}$	$7.71\cdot10^{-4}$	$1.91\cdot10^{-2}$
4(b)	$1.36\cdot10^{-3}$	$4.67\cdot10^{-4}$	$1.33\cdot10^{-3}$	$3.37\cdot10^{-2}$
4(c)	$7.01\cdot10^{-5}$	$1.41\cdot10^{-5}$	$6.24\cdot10^{-4}$	$8.65\cdot10^{-3}$
4(d)	$5.73\cdot10^{-5}$	$1.66\cdot10^{-5}$	$1.17\cdot10^{-3}$	$9.34\cdot10^{-3}$
4(e)	$1.11\cdot10^{-2}$	$4.00\cdot10^{-4}$	$6.17\cdot10^{-2}$	$2.08\cdot10^{-2}$
5(a)	$2.89\cdot10^{-4}$	$2.48\cdot10^{-5}$	$7.31\cdot10^{-4}$	$1.43\cdot10^{-2}$
5(b)	$1.66\cdot10^{-4}$	$6.25\cdot10^{-5}$	$1.02\cdot10^{-4}$	$2.05\cdot10^{-2}$
6	$8.89\cdot10^{-5}$	$1.69\cdot10^{-5}$	$1.77\cdot10^{-3}$	$1.13\cdot10^{-2}$

Table S1. The one standard deviation errors of the nonlinear curve fits for the various configurations, where the units of the output signal is volts.

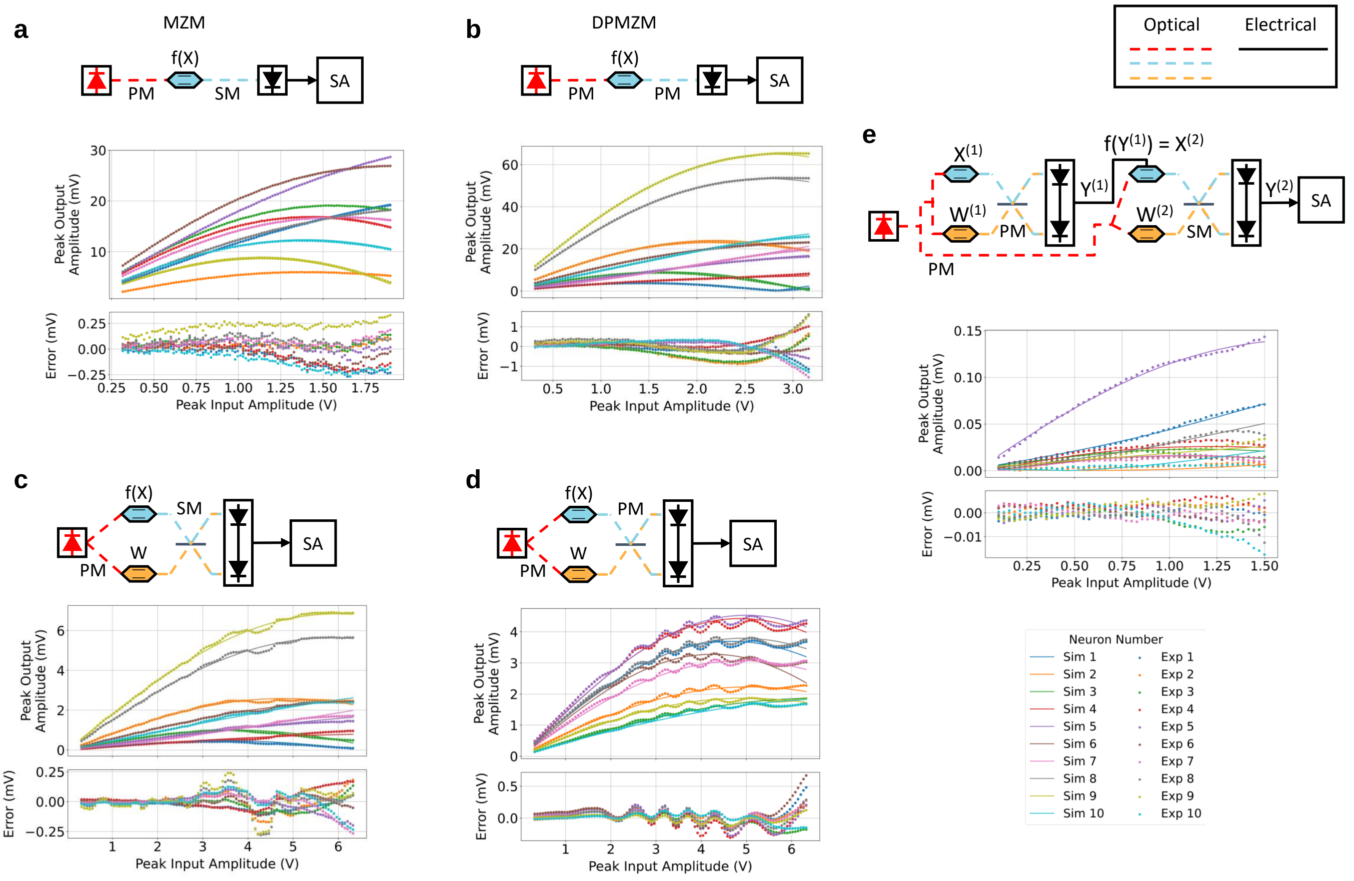

Figure S4. Various configurations for characterizing the MZM nonlinearity.

The input vector signal $V_X(t)$ contained 10 frequencies from 100 MHz to 109 MHz spaced at 1 MHz. A single (non-balanced) photodetector was used here.

Figure S4(b) has the same configuration as that in Figure S4(a), except the MZM is replaced with a DPMZM with a PM output fiber lead. For all configurations with DPMZMs, we characterize the nonlinearity by driving only one of the sub-MZMs. In this case, we used the bias controller to set the DPMZM to the SSB-SC mode that sets both sub-MZMs to their minimum bias point. Then we manually changed the bias point of the sub-MZM under test to quadrature. The frequencies of $V_X(t)$ are the same as in S4(a), but with a different set of randomized values. This is the nonlinear characterization shown in the main text.

Figure S4(c) uses an interferometer configuration with two DPMZMs, testing the nonlinear behavior with photoelectric multiplication. Here, the DPMZMs have an SM fiber output lead, and thus all fibers after the DPMZMs are SM. As explained in Supplementary Section A, all sub-MZMs were biased at their minimum point. Here, $V_X(t)$ is a 10-frequency signal from 10 MHz to 19 MHz spaced at 1 MHz with randomized values. And similar to the linear characterization, we set $V_W(t)$ to be a single tone at 50 MHz, measuring the output signal from 60 MHz to 69 MHz.

Figure S4(d) has the same hardware configuration and parameters as (c), except that we used DPMZMs with PM fiber output leads, and thus used PM fibers throughout the whole configuration. We consistently observed a ripple with this configuration, which is discussed later with Figure S6. This is same the configuration used for the linear characterization in Supplementary Section B (excluding the RF amplifier).

Figure S4(e) is the same configuration as the 3-layer experiment in the main text. This figure shows the curve fit that was used to infer the 10,000 MNIST images. The SA measurements for both the curve fit and the MNIST inference experiment in the main text were single-shot readouts without averaging. For this curve fit, the values of $X^{(1)}$, $W^{(1)}$, and $W^{(2)}$ were all randomized. For the MNIST inference and DNN training, the nonlinearity was programmed at the strongest setting (the right-most set of data points).

Figure S5 repeats the measurement for two of the configurations from Figure S4, except setting $V_X(t)$ to be a 49-frequency signal (the same size as a downsampled 7×7 MNIST image) from 10.1 MHz to 14.9 MHz with 100 kHz spacing, with randomized values. The configuration in Figure S5(b) again sets $V_W(t)$ to be a single

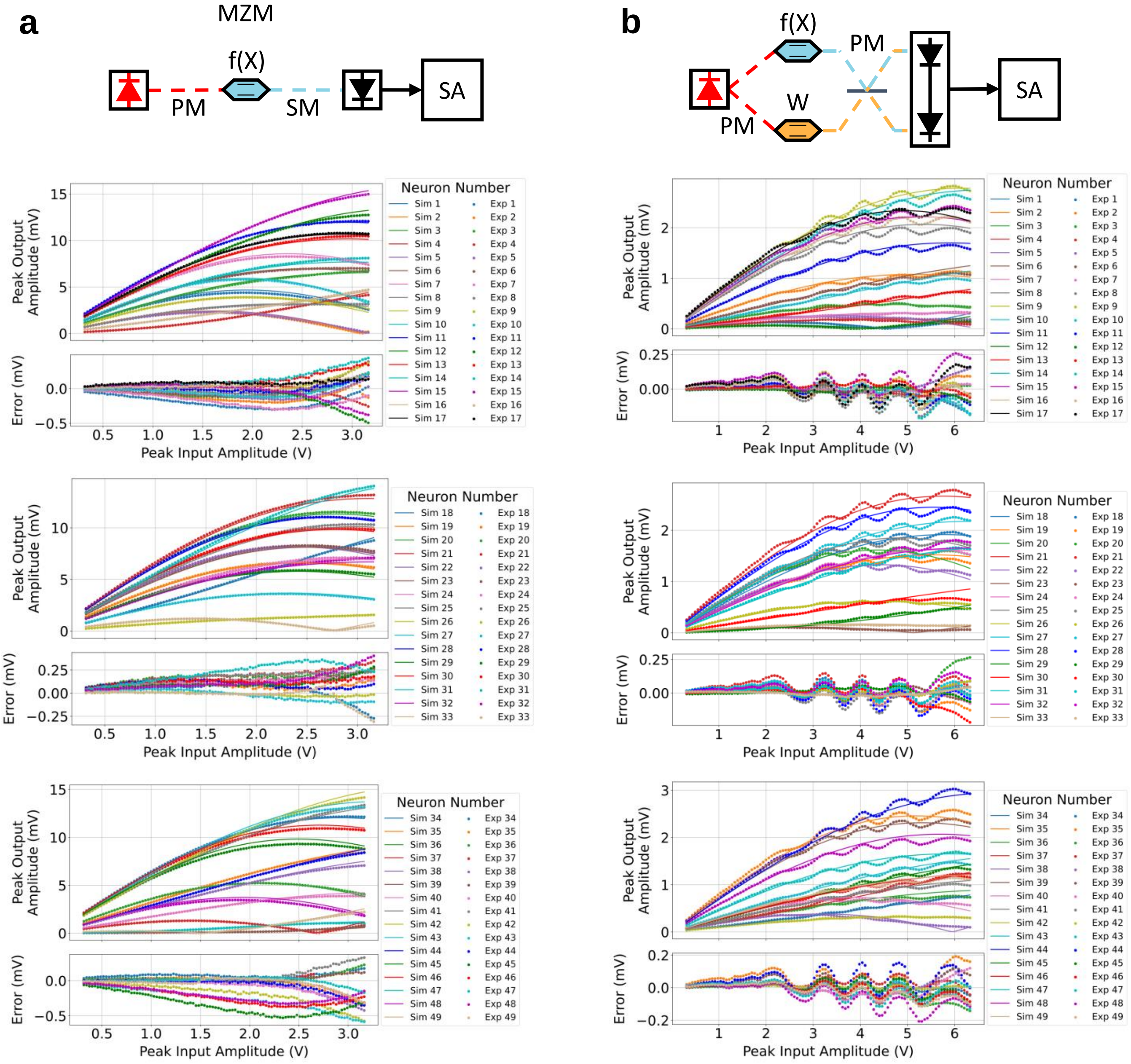

Figure S5. A 49-frequency (size of an downsampled 7 × 7 MNIST image) nonlinear characterization, where we plot the nonlinear curves of all 49 neurons for two configurations from Figure S4.

tone at 50 MHz. We plot the nonlinear curves of all 49 neurons, thus demonstrating a larger scale nonlinearity.

Figure S6 explores the ripples observed in the nonlinear characterization of the hardware configuration in Figure S4(d). Here, the curve fit was only applied to the top left measurement, and the same parameters were used for the other three measurements. All four measurements were taken within 40 minutes. Here, we see that the number and position of the ripples vary per measurement. We hypothesize that the ripples are caused either by (i) interference due to imperfect attenuation of the laser carrier and sideband that allows for SSB-SC modulation or (ii) optical path length imbalance in the interferometer. If the cause is (i), then this can perhaps be rectified by achieving SSB-SC modulation using a passive optical filter or a different bias control scheme. For (ii), then introducing efforts to balance the optical paths may help.

Modulation Classification: The curve-fit for the 3-layer modulation classification used a similar curve fit method but a slightly different setup. Here five curve fit parameters were used:

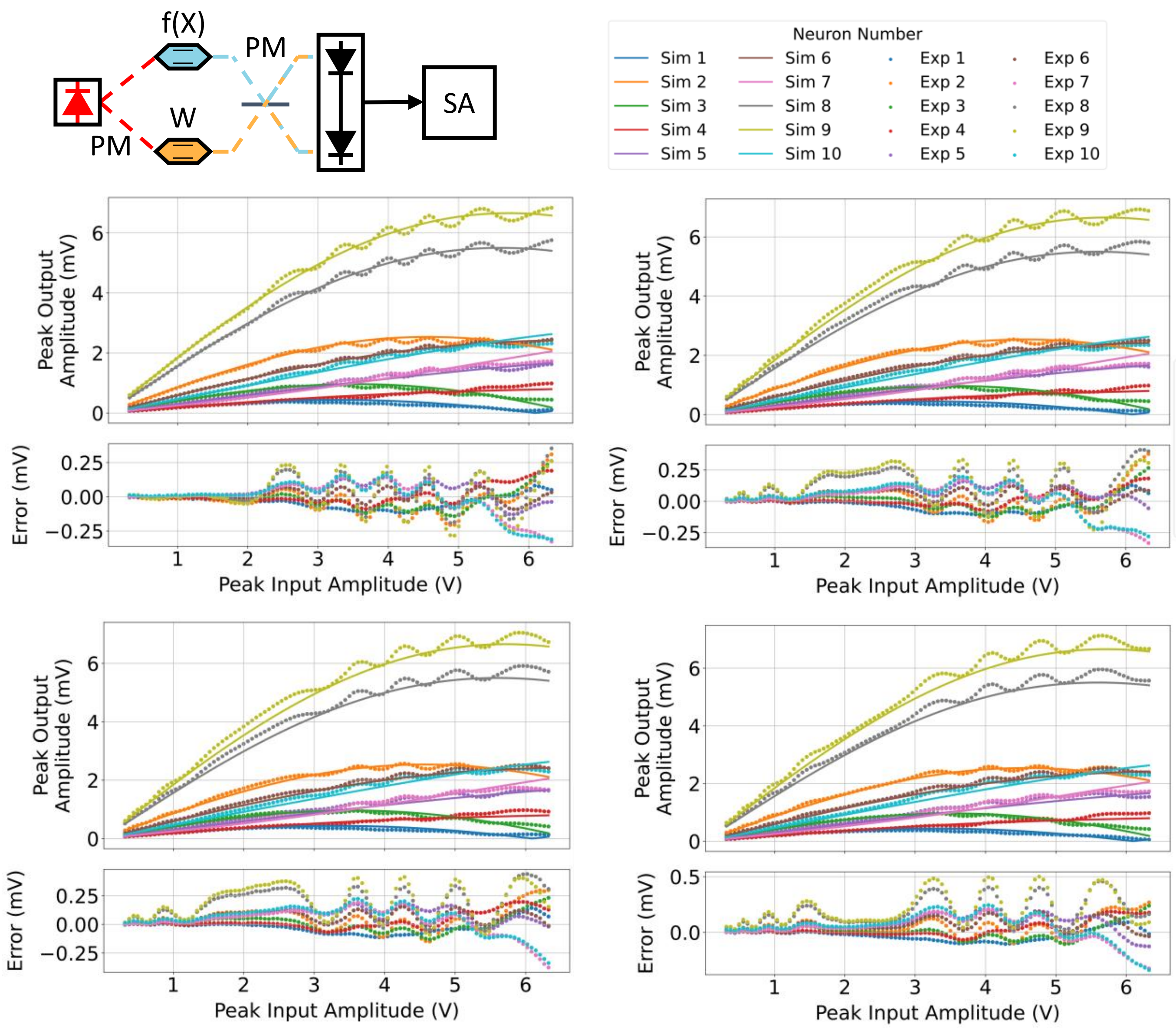

Figure S6. A nonlinear measurement that was repeated four times to explore the nature of the ripples observed in Figure S4(d). The curve fit parameters are the same for all four experiments and were derived from the top left measurement.

$$V_{\text{out}}^{(1)}(t) = \chi_0 + \chi_1 \sin\left(\chi_2 V_X^{(1)}(t) + \chi_3\right)$$
$$V_{\text{out}}^{(2)}(t) = \chi_4 \text{Im}\left[\left(E_X^{(2)}(t)\right)^* E_W^{(2)}(t)\right].$$

Using the new parameter χ_4 does not introduce any new free variables in the curve fitting model but does enable the curve fitting algorithm to potentially find a more realistic fit. The curve fitting parameters used in the modulation classification experiment in main text Figure 3D are: $\chi_0 = 2.832$ with 1-STD error 550,320; $\chi_1 = 1.097$ with 1-STD error 213,189; $\chi_2 = -3.409$ with 1-STD error 0.161; $\chi_3 = 0.236$ with 1-STD error 0.049; and $\chi_4 = 0.409$ with 1-STD error 79,569.

In addition instead of curve-fitting using the increasing-amplitude versus frequency mode magnitude curve as in the other nonlinear figures, we curve fitted the parameters using the FFT of $V_{\text{out}}^{(2)}(t)$ itself. Although the curve fit parameters were accurate enough to achieve 85.0% experimental accuracy for modulation classification, the high errors suggest that there may be a device imperfection or a gap in the physics model of the hardware.

D. Spectrum Control with Matrix Operations

The 2×2 matrix analysis in the main text conveyed one scheme of programming a matrix-vector multiplication with MAFT. However, MAFT also offers flexibility in determining the output spectrum of the matrix-vector products. Figure S7 shows various methods of programming the spectrum of the output.

In this example, $V_X(t)$ contains two frequencies, $V_W(t)$ contains four frequencies, and the output electrical voltage signal $V_{\text{out}}(t)$ contains a variable number of frequencies. We keep $V_X(t)$ the same while altering $V_W(t)$ to demonstrate various effects. In Figure S7(b), $V_W(t)$ is programmed so that each partial sum in $V_{\text{out}}(t)$ maps to a unique frequency, thus performing no summation in the frequency domain but instead performing a 1D convolution. In Figure S7(c), $V_W(t)$ is programmed so that the frequency domain of $V_{\text{out}}(t)$ yields a matrix-vector product where the frequencies corresponding to the elements of Y are adjacently spaced in the middle of the spurious frequencies. We refer to this method of programming as our *frequency reduction scheme* because $\Delta\omega_Y < \Delta\omega_X$. Figure S7(d) demonstrates an alternative method of programming $V_W(t)$, which intersperses the elements of Y with the spurious frequency components. We term this the *frequency expansion scheme*, as $\Delta\omega_Y > \Delta\omega_X$. The frequency reduction and expansion schemes can be used alternatively for consecutive layers of a DNN to avoid running out of bandwidth.

Each partial sum term in $V_{\text{out}}(t)$ in Figures S7(b)-(d) can be traced to difference between the input and weight frequencies. For example, in Figure S7(b) the partial sum term $W_{22}X_1$ derives from the product of W_{22} at 9 MHz and X_1 at 1 MHz, where the difference between their frequencies causes $W_{22}X_1$ to appear at 8 MHz.

E. Offline Physics-Based DNN Training

For the 3-layer DNN MNIST inference experiment, the DNN was trained offline using an analytic model of the hardware. As explained in Supplementary Section C, four parameters were curve fitted to the experimental hardware. One challenge was to create a DNN training algorithm that accurately models the photoelectric multiplication and nonlinearity while being fast enough to train the DNN in a reasonable time.

Pytorch was used for the DNN training, as the software automatically calculates the gradients for training the parameters, as long as only Pytorch functions are used to model the physics. Algorithm 1 shows the pseudocode to quickly model the physics of the system. We briefly explain each of the steps:

Algorithm 1: Physics-Based DNN Training

1: $\Delta f \leftarrow \text{GCD(List of Expected Frequencies)}$
2: $\widetilde{X}^{(1)}(f), \widetilde{W}^{(1)}(f), \widetilde{W}^{(2)}(f) \leftarrow \text{DNN Matrix Values}$
3: $X^{(1)}(t), X^{(1)}_{\text{shift}}(t) \leftarrow \text{DCT}(\widetilde{X}^{(1)}(f)), \text{DCT}_{\text{variant}}(\widetilde{X}^{(1)}(f))$
4: $W^{(1)}(t), W^{(1)}_{\text{shift}}(t) \leftarrow \text{DCT}(\widetilde{W}^{(1)}(f)), \text{DCT}_{\text{variant}}(\widetilde{W}^{(1)}(f))$
5: $W^{(2)}_{\text{shift}}(t) \leftarrow \text{DCT}_{\text{variant}}(\widetilde{W}^{(2)}(f))$
6: $Y^{(1)}(t) \leftarrow X^{(1)}(t)W^{(1)}_{\text{shift}}(t) - X^{(1)}_{\text{shift}}(t)W^{(1)}(t)$
7: $f(Y^{(1)}(t)) \leftarrow \chi_0 + \chi_1 \sin\left(\chi_2 Y^{(1)}(t) + \chi_3\right)$
8: $X^{(2)}(t) \leftarrow f(Y^{(1)}(t))$
9: $Y^{(2)}(t) \leftarrow X^{(2)}(t)W^{(2)}_{\text{shift}}(t)$
10: $\widetilde{Y}^{(2)}(f) \leftarrow \text{DCT}(Y^{(2)}(t))$

1. Taking the greatest common denominator (GCD) of some frequencies we expect to be present to discretize the frequency space
2. Directly inserting the vector and matrix values into the frequency domain
3. Converting the inputs to the time domain, where the "shift" of the signal is the 90° shifted signal used for SSB-SC modulation, and the "variant" of the discrete cosine transform (DCT) is an altered version of the DCT that yields the desired 90° shifted signal
4. Converting the layer 1 weights to the time domain
5. Converting the layer 2 weights to the time domain
6. The linear output of the first photoelectric multiplication; this signal contains the spurious frequencies, which can be filtered out at this point if desired
7. Applying the curve fitted nonlinear activation
8. The nonlinear output of the first layer is the input to the second layer
9. The second photoelectric multiplication, modeling the photoelectric multiplication of the input in the DSB-SC mode and the weights in the SSB-SC mode
10. Converting the output to the frequency domain to retrieve the neuron values

We also included the effects of the AWG scaling and the frequency correction mentioned in Supplementary Section B into the algorithm, depending on the hardware configuration being modeled. And although we did not use any frequency filters for our experiments, we incorporated the effects of attenuation from a filter into the DNN model.

The physics-based DNN training was found to take up to approximately 5-6 times longer to train compared to a conventional DNN, depending on the workload. A better optimized physics-based algorithm should be able to cut the time down.

Note that this algorithm avoids using complex numbers to model the hardware or train the DNN. However, the training for the modulation classification used a similar algorithm but one that included complex numbers so that both the magnitudes and phases of the weight signal could be trained. Hence, the algorithm above is useful

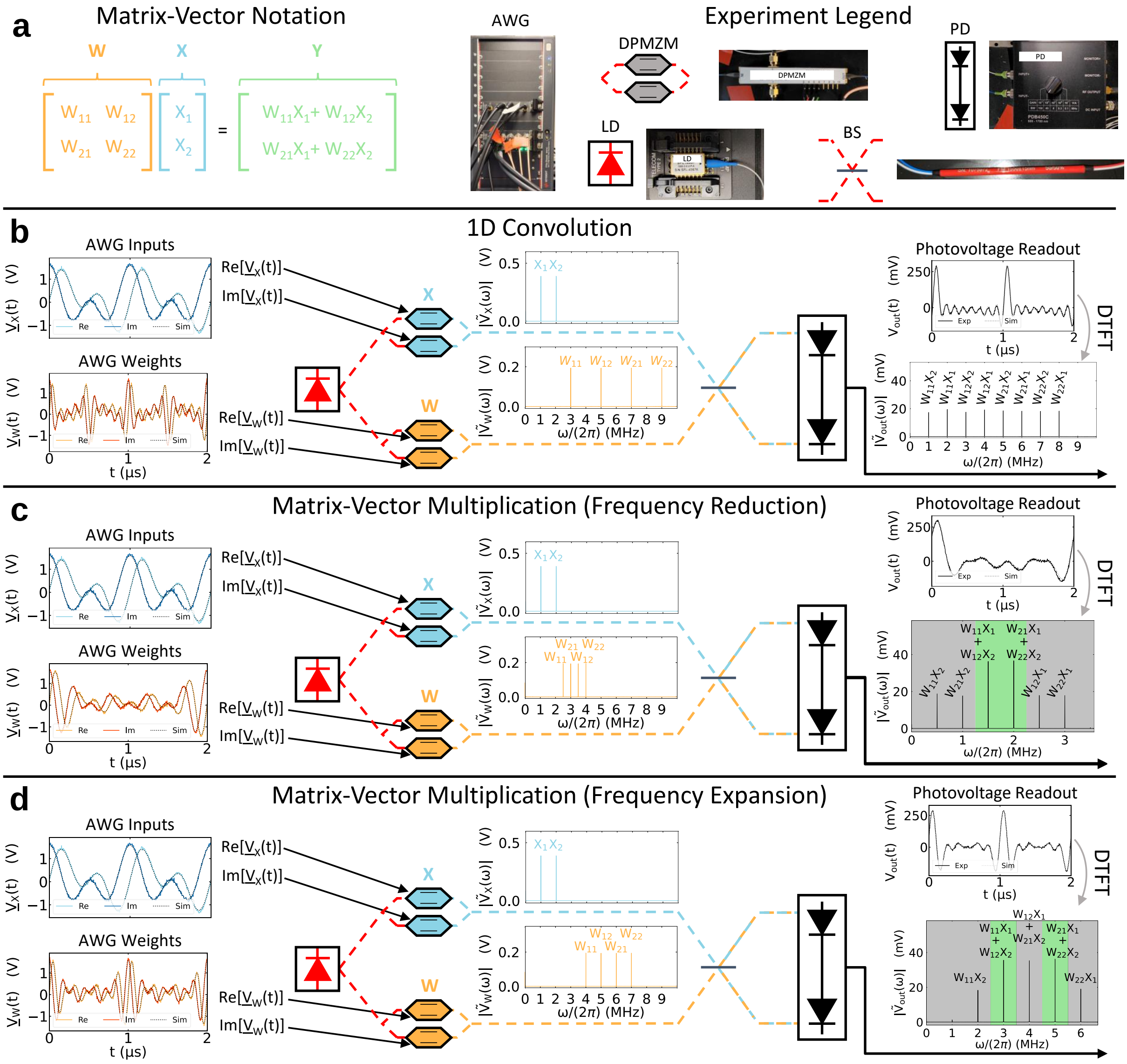

Figure S7. An experimental example of various methods of computing using the MAFT scheme. Each time domain plot shows one period of the raw data from the oscilloscope, and each frequency domain plot shows the discrete time Fourier transform (DTFT) of the entirety of the oscilloscope trace. (a) The notation for a typical 2×2 matrix multiplication between a matrix W and a vector X. The legend contains photos of the commercial components used to conduct the experiments in this figure. (b) 1D Convolution. In this case, the frequencies of $V_W(t)$ were chosen to demonstrate a separation of the eight partial sums contained in the frequencies of $V_{\text{out}}(t)$. (c) Frequency reduction scheme. $V_W(t)$ is programmed to yield a matrix-vector product where $\Delta\omega_Y < \Delta\omega_X$. Here, the spurious frequencies (gray region) are pushed to either side of the neuron frequencies. The unique partial sum terms contained in the spurious frequencies of $V_{\text{out}}(t)$ will either be removed with a bandpass filter or used to train a DNN. (d) Frequency expansion scheme. Here, $V_W(t)$ is programmed so that $\Delta\omega_Y > \Delta\omega_X$. As with the previous scheme, the spurious frequencies in $V_{\text{out}}(t)$ will either be removed with a periodic filter or used to train the DNN. The two frequency encoding schemes can be used alternatively to avoid bandwidth limitations for arbitrarily deep DNNs.

for scenarios where complex numbers are best avoided when training the DNN.

F. LTI Experiments

The three LTI signal processing experiments used the hardware setup shown in Figure S4(d) (though operating in the linear regime of the DPMZMs). As explained in

Supplementary Section A, each DPMZM has three DC biases. To control whether the left or right sideband suppressed in SSB-SC operation, we programmed the polarity of the quadrature setting (positive versus negative quadrature) on the bias controller. (The 'left' sideband is the sideband with frequency lower than the laser carrier and the 'right' sideband has higher frequency than the laser carrier.)

The LTI experiments require the phase information in the final readout of the FFT of the output waveform so that we can distinguish between the positive and negative frequencies. Therefore instead of using a spectrum analyzer or taking the magnitude of the FFT from an oscilloscope, we took the imaginary part of the FFT to retain the phase information. We inferred the correct timing of each waveform with a time domain matched filter and by observing the gradient and location of the phases (as a time delay deterministically introduces a phase gradient).

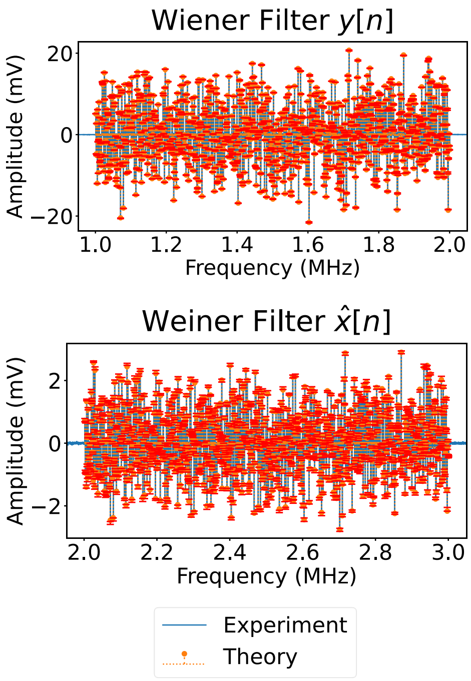

Figure S8. Example experimental LTI measurements. Each plot overlays the experiment and the theoretical signal in the Wiener filter scenario. The top measurement compares the experimental generation of $y[n]$ from the AWG to the theoretical signal. The bottom measurement compares the output of MAFT-ONN of $\hat{x}[n]$ to the theoretical signal. The experimental waveform plotted is the mean of 10 experiments and the red error bars are the standard deviation of those experiments.

Each LTI experimental waveform was repeated 10 times on the MAFT-ONN hardware. The MSEs were calculated by normalizing the experimental LTI waveforms with the theoretical LTI signals to account for the output scaling due to MAFT-ONN physics, ensuring that the signals were properly compared. In main text Figures 3A-C, the signals $x[n]$, $y[n]$, $h[n]$, and $x_{\text{target}}[n]$ were measured directly from the AWG while the signals $\hat{x}[n]$, $x_1[n]$, $x_2[n]$, and $x_3[n]$ were measured outputs of MAFT-ONN. Figure S8 shows an example of an overlay of the theoretical versus experimental LTI signals where the experimental waveform is the mean of 10 experiments and the error bars are the standard deviation.

THEORY

G. General MAFT Algorithm

This general frequency-encoding algorithm will show that a set of input frequencies can be transformed to an arbitrary set of output frequencies while computing an arbitrary matrix-vector product $WX = Y$, where W has size $(R \times N)$, X has size $(N \times 1)$, and Y has size $(R \times 1)$. This is all accomplished in a single shot in the frequency domain.

We assume that the input activation signal has frequency spacing $\Delta\omega_X$ and offset $n_0 \cdot \Delta\omega_X$; hence, the input vector begins as the electrical voltage signal $V_X(t) = \sum_{n=1}^{N} X_n \cos((n_0+n)\Delta\omega_X t)$. Here we assume equal frequency spacing for the input signal for convenience, without loss of generality. The input signal can express an arbitrary set of frequencies by decreasing $\Delta\omega_X$ and setting the non-occupied frequencies to zero. Now we do not assume anything about the output signal frequencies, leaving the weight frequencies as arbitrary values. Therefore, let the frequency corresponding to the weight matrix element $W_{r,n}$ be $\omega^W_{r,n}$. This yields the electrical voltage signal for the weight matrix: $V_W(t) = \sum_{r=1}^{R}\sum_{n=1}^{N} W_{r,n}\cos(\omega^W_{r,n} t)$.

Next, $V_X(t)$ and $V_W(t)$ are SSB-SC modulated onto a laser carrier with frequency ω_{LD}. This yields (ignoring linear scaling factors):

$$E_X(t) \propto \sum_{n=1}^{N} X_n e^{i((n_0+n)\Delta\omega_X + \omega_{LD})t},$$

$$E_W(t) \propto \sum_{r=1}^{R}\sum_{n=1}^{N} W_{r,n} e^{i(\omega^W_{r,n} + \omega_{LD})t}.$$

Next, the optical fields $E_X(t)$ and $E_W(t)$ enter a 2×2 50:50 beam splitter and then a balanced photodetector for the photoelectric multiplication. The optical outputs of the beam splitter are:

$$E_{\text{BS1}}(t) \propto E_X(t) - iE_W(t),$$
$$E_{\text{BS2}}(t) \propto -iE_X(t) + E_W(t).$$

Then $E_{\text{BS1}}(t)$ and $E_{\text{BS2}}(t)$ each enter a photodetector, and the outputs are subtracted to yield the photoelectric multiplication output (again ignoring scaling factors):

$$
\begin{aligned}
V_{\text{out}}(t) &\propto |E_{\text{BS1}}(t)|^2 - |E_{\text{BS2}}(t)|^2 \\
&\propto \text{Im}\left[E_X^*(t) E_W(t)\right] \\
&\propto \text{Im}\left[\sum_{r=1}^{R}\sum_{n'=1}^{N}\sum_{n=1}^{N} W_{r,n'} X_n e^{i((\omega^W_{r,n'} - (n_0+n)\Delta\omega_X)t)}\right] \\
&\propto \sum_{r=1}^{R}\sum_{n'=1}^{N}\sum_{n=1}^{N} W_{r,n'} X_n \sin((\omega^W_{r,n'} - (n_0+n)\Delta\omega_X)t)
\end{aligned}
\tag{S1}
$$

Everything so far in this analysis has only considered the physics of the system. Now we can choose the interpretation of the signals to represent matrix-vector product. By definition of matrix multiplication, the output matrix elements are: $Y_r = \sum_{n=1}^{N} W_{r,n} X_n$. By comparing this definition of a matrix product to Equation S1, we see that only the frequencies that correspond to $n' = n$ will contribute to the desired operation. All other frequencies will be spurious. There are a total of RN^2 unique partial sums generated by this photoelectric multiplication.

This matrix-vector product can be achieved by programming the weight frequencies to group some of the RN^2 partial sums into R groups of N frequencies. Each group is associated with a value r and thus the element of the output matrix Y_r. Say that we want to map each output matrix element Y_r to frequency ω_r^Y. Then the solution for the weight matrix frequencies to achieve this desired frequency transformation is:

$$
\omega^W_{r,n'} = \omega_r^Y + (n_0 + n')\Delta\omega_X, \tag{S2}
$$

for $n' \in [1, \ldots, N], r \in [1, \ldots, R]$. Plugging Equation S2 into Equation S1, we have:

$$
V_{\text{out}}(t) \propto
$$

$$
\propto \sum_{r=1}^{R}\sum_{n'=1}^{N}\sum_{n=1}^{N} W_{r,n'} X_n \sin((\omega_r^Y + (n'-n)\Delta\omega_X)t) \tag{S3}
$$

$$
\begin{aligned}
&\propto \sum_{r=1}^{R}\sum_{n=1}^{N} W_{r,n} X_n \sin(\omega_r^Y t) \\
&\qquad + \sum_{r=1}^{R}\sum_{n'=1}^{N}\sum_{\substack{n=1 \\ n\neq n'}}^{N} W_{r,n'} X_n \sin((\omega_r^Y + (n'-n)\Delta\omega_X)t)
\end{aligned}
$$

$$
\propto \sum_{r=1}^{R} Y_r \sin(\omega_r^Y t) \tag{S4}
$$

$$
\begin{aligned}
&\qquad + \sum_{r=1}^{R}\sum_{n'=1}^{N}\sum_{\substack{n=1 \\ n\neq n'}}^{N} W_{r,n'} X_n \sin((\omega_r^Y + (n'-n)\Delta\omega_X)t) \\
&\propto V_Y(t) + V_S(t),
\end{aligned}
\tag{S5}
$$

where we ignore the linear scaling factor, which is discussed in Supplementary Section B. From Equation S4 to Equation S5, we group the terms with $n' = n$ as the output vector Y, described as $V_Y(t)$ and the terms with $n' \neq n$ as the 'spurious frequency' components $V_S(t)$.

Thus, by programming the weight signal accordingly, we perform both a matrix-vector product and frequency transformation. The electric voltage output signal is $V_Y(t) = \sum_{r=1}^{R} Y_r \sin(\omega_r^Y t)$, and the remainder of the signal contains the spurious frequencies. We arrive to the same results in the main text by choosing equally spaced output vector frequencies: $\omega_r^Y = (r_0 + r)\Delta\omega_Y$.

H. Anti-Aliasing Conditions

When computing matrix-vector products for a fully connected layer, the frequency reduction and expansion schemes from Supplementary Section D use the result from Equation S4 in a way that prevents aliasing. This occurs when some of the spurious frequencies overlap with the output vector frequencies. Each scheme avoids aliasing in a different way. Recall that in both schemes, $\omega_r^Y = (r_0 + r)\Delta\omega_Y$.

First, we analyze the anti-aliasing conditions of the frequency reduction scheme. Here, the frequency spacing of the output frequencies are less than that of the input frequencies: $\Delta\omega_Y < \Delta\omega_X$. As Figure S9 illustrates, there are two types of aliasing that must be avoided.

The first type of aliasing, shown in Figure S9(a), originates from the overlapping of the spurious frequencies with the output vector frequencies. We find the anti-aliasing constraint by keeping track of the distance of the closest spurious frequency to the edge of band of output vector frequencies. From Equation S4, the lowest spurious frequency after the band is $(r_0 + 1)\Delta\omega_Y + \Delta\omega_X$, and the highest output vector frequency is $(r_0 + R)\Delta\omega_Y$. Thus, as labeled in Figure S9(a) the gap between these frequencies is $\Delta\omega_X + (1 - R)\Delta\omega_Y$. Hence, for anti-aliasing, this gap must be greater than 0 at minimum. This yields the constraint: $\Delta\omega_Y < \frac{1}{R-1}\Delta\omega_X$. A reasonable design choice, which is the one we made for the experiment, is to set this gap equal to the smallest frequency spacing already present in the signal, $\Delta\omega_Y$. So with this anti-aliasing condition we get: $\Delta\omega_Y = \frac{1}{R}\Delta\omega_X$.

The second anti-aliasing condition comes from the fact that the signal is real, and thus, in the frequency domain, it is possible for the negative part of the signal to creep into the positive region. This phenomenon is conveyed in Figure S9(b). Here, we calculate the gap between the lowest output vector frequency and the least-negative spurious frequency. The former is $(r_0 + 1)\Delta\omega_Y$. The latter, according to Equation S4, is $-((1 - N)\Delta\omega_X + (r_0 + 1)\Delta\omega_Y)$. Therefore, as labeled in Figure S9(b) the frequency gap is $2(r_0 + 1)\Delta\omega_Y - (N - 1)\Delta\omega_X$. Again, we want this gap to be greater than 0 to avoid aliasing, so we end up with the constraint $(r_0 + 1)\Delta\omega_Y > \frac{1}{2}(N - 1)\Delta\omega_X$. Another reason-

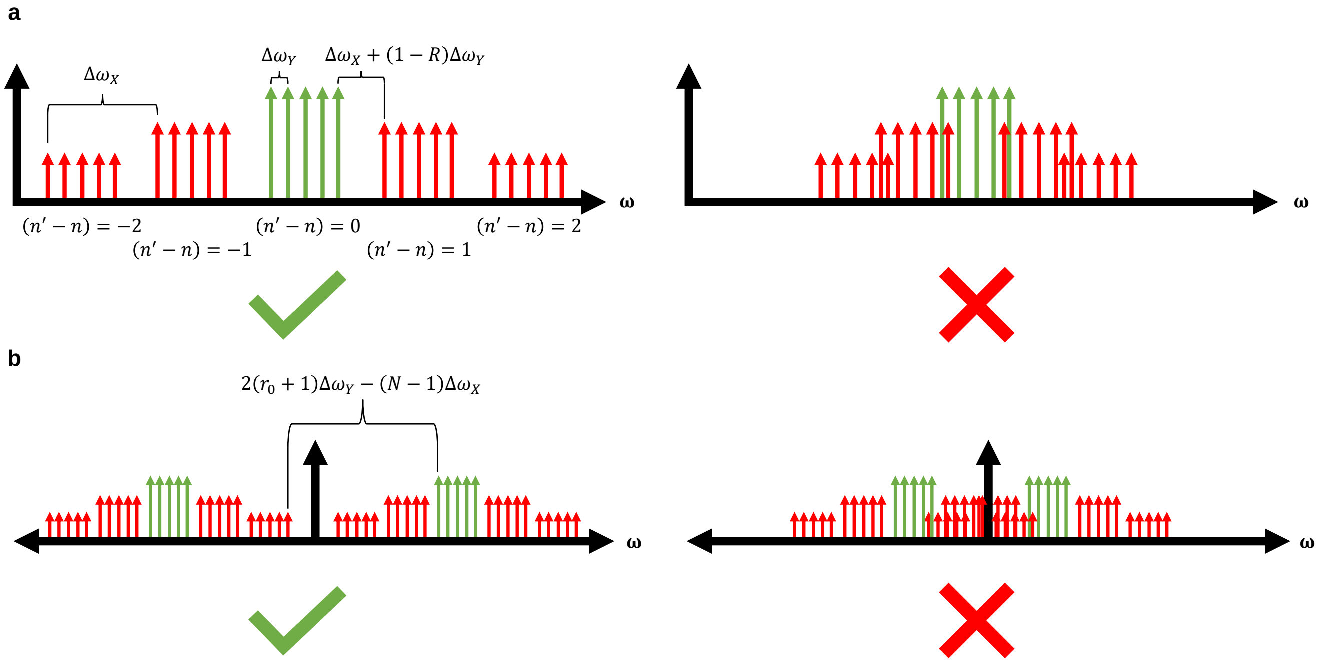

Figure S9. The anti-aliasing conditions for the frequency reduction scheme, where the output vector frequencies are green and the spurious frequencies are red. (a) The first type of aliasing comes from the spurious frequencies overlapping with the output vector frequencies. This can be avoided by decreasing the output frequency spacing. (b) The second type of aliasing comes from the negative frequencies creeping up to the positive region. This can be avoided by increasing output frequency offset.

able design choice is to also set this gap equal to $\Delta\omega_Y$. Applying both design choices, we solve for the term r_0 to get: $r_0\Delta\omega_Y = \frac{1}{2}(N - \frac{R+1}{R})\Delta\omega_X$. Now we have a set of constraints and a way to program the weight signal frequencies to guarantee that the spurious frequencies do not overlap with the output vector frequencies when using the frequency reduction scheme.

Next is the frequency expansion scheme, where the output vector frequency spacing is larger than the input frequency spacing; $\Delta\omega_Y > \Delta\omega_X$. Figure S10 illustrates the anti-aliasing conditions. The two types of aliasing here are the same as in the frequency reduction scheme.

For the first type of aliasing, showed in Figure S10(a), we calculate the frequency gap between the jth output vector frequency and the closest spurious frequency. The frequency of the jth output vector is $(r_0 + j)\Delta\omega_Y$, and the closest spurious frequency is $(r_0 + j - 1)\Delta\omega_Y + (N - 1)\Delta\omega_X$. (The spurious frequencies are symmetric about each output vector frequency, so this calculation can also be done with the higher spurious frequency.) Thus, the frequency gap is $\Delta\omega_Y - \Delta\omega_X(N - 1)$. Therefore, the anti-aliasing condition is: $\Delta\omega_Y > \Delta\omega_X(N - 1)$. And another reasonable design choice is to set this gap equal to the smallest frequency spacing present in the signal, $\Delta\omega_X$. With this, we get: $\Delta\omega_Y = N\Delta\omega_X$.

Figure S10(b) shows the second type of aliasing for the frequency expansion scheme, where the negative frequencies of the real signal creep into the position region. The constraint arising from this aliasing is derived in the same way. The lowest output vector frequency is $(r_0 + 1)\Delta\omega_Y$, and the least-negative spurious frequency is $-((1 - N)\Delta\omega_X + (r_0 + 1)\Delta\omega_Y)$. Thus, the frequency gap is $2(r_0 + 1)\Delta\omega_Y - (N - 1)\Delta\omega_X$, yielding the anti-aliasing constraint: $(r_0 + 1)\Delta\omega_Y > \frac{1}{2}(N - 1)\Delta\omega_X$. Interestingly, when using the design choice from above, this constraint becomes $r_0\Delta\omega_Y > -\frac{1}{2}(N - 1)\Delta\omega_X$. Since the constraint is a negative number, we can simply set the offset frequency for the output vectors to zero as a convenient design choice, so $r_0 = 0$.

As shown above, the spurious frequencies generated by the photoelectric multiplication requires anti-aliasing conditions to ensure they do not degrade the matrix-vector product. In addition to the constraints, we also presented some convenient design decisions for handling the spectrum. These design decisions do not have to be followed, however. For example, for our experiment, in the layer 1 frequency reduction scheme, we chose a value of r_0 that was larger than necessary in order to push all the frequencies beyond 2 MHz, which is the lower limit of our RF amplifier.

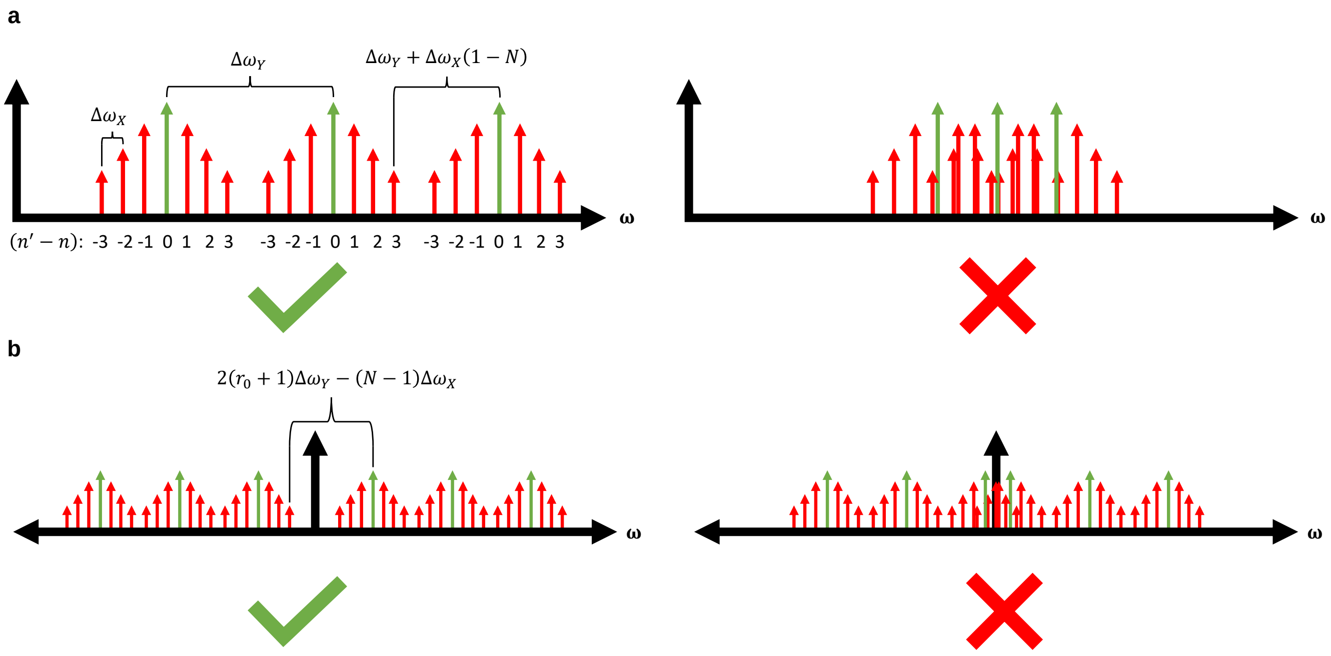

Figure S10. The anti-aliasing conditions for the frequency expansion scheme, where the output vector frequencies are green and the spurious frequencies are red. (a) The first type of aliasing comes from the spurious frequencies overlapping with the output vector frequencies. This can be avoided by increasing the output frequency spacing. (b) The second type of aliasing comes from the negative frequencies creeping up to the positive region. This can be avoided by increasing output frequency offset.

I. Throughput Derivation

The throughput is a measure of the volume of data that can be processed within a given time. Since this architecture directly computes matrix-vector products, we analyze the throughput of a single fully-connected layer of a DNN. From the main text, the general throughput equation is:

$$T = \frac{\#\text{ MACs}}{\text{latency}} = N \cdot R \cdot \min(\Delta f, f_0), \tag{S6}$$

where N is the number of input neurons into the layer, R is the number of output neurons, Δf is the smallest spacing of the output signal, and f_0 is the lowest neuron frequency of the output signal. Here we derive the throughput for the frequency reduction and expansion schemes, given the anti-aliasing conditions found in the previous section.

The available bandwidth with which to modulate the inputs and weights, B, limits the weight matrix signal, as that signal will always have a higher frequency than the input signal, as shown in Equation S2. Therefore, the maximum bandwidth used to modulate the signals is: $B = (n_0 + N)\Delta f_X + (r_0 + R)\Delta f_Y$. As we saw in Supplementary Section G, the term n_0 cancels out and does not affect the output frequencies. So as a design choice to maximize the available bandwidth of the components, we set $n_0 = 0$ to yield $B = N\Delta f_X + (r_0 + R)\Delta f_Y$.

For the frequency reduction scheme, it is clear that the limiting factor in the term $\min(\Delta f, f_0)$ is the frequency spacing of the output signal, Δf_Y. This is because by definition the frequency reduction scheme requires that $\Delta f_Y < \Delta f_X$, and we see from the anti-aliasing conditions that $\Delta f_Y < r_0 \Delta f_Y$. Hence, the throughput of the frequency reduction scheme is: $T_{\text{reduction}} = N \cdot R \cdot \Delta f_Y$.

Now we can solve for the throughput in terms of the bandwidth, using the design choices mentioned in the anti-aliasing section, as those choices maximize the throughput. First, we can plug in the value of Δf_Y into the throughput equation using the anti-aliasing result $\Delta f_Y = \frac{1}{R}\Delta f_X$ to yield: $T_{\text{reduction}} = N \cdot \Delta f_X$.

Dividing the throughput by the bandwidth, we get:

$$\frac{T_{\text{reduction}}}{B} = \frac{N\Delta f_X}{N\Delta f_X + (r_0 + R)\Delta f_Y}.$$

Here, we can again plug in the anti-aliasing values of $\Delta f_Y = \frac{1}{R}\Delta f_X$ and $r_0 \Delta f_Y = \frac{1}{2}(N - \frac{R+1}{R})\Delta f_X$, to yield:

$$\begin{aligned}
&\frac{N\Delta f_X}{N\Delta f_X + (r_0+R)\Delta f_Y} = \\
&= \frac{N\Delta f_X}{N\Delta f_X + \frac{1}{2}\left(N - \frac{R+1}{R}\right)\Delta f_X + \frac{\Delta f_X}{\Delta f_Y}\Delta f_Y} \\
&= \frac{N\Delta f_X}{N\Delta f_X + \frac{1}{2}\left(N - \frac{R+1}{R}\right)\Delta f_X + \Delta f_X} \\
&= \frac{N}{N + \frac{1}{2}\left(N - \frac{R+1}{R}\right) + 1} \\
&= \frac{2NR}{3NR + R + 1},
\end{aligned}$$

where the final equality came from simplifying the expression. To get the final approximation for the throughput, we can take the reciprocal of this expression to see that:

$$\begin{aligned}
\frac{B}{T_{\text{reduction}}} &= \frac{3NR + R + 1}{2NR} \\
&= \frac{3}{2} + \frac{1}{2N} - \frac{1}{2NR} \\
&\approx \frac{3}{2}, \quad \text{for} \quad N \gg 1.
\end{aligned}$$

Finally, taking the reciprocal again, we have both the exact and approximate expressions for the throughput of the frequency reduction scheme under the anti-aliasing conditions:

$$T_{\text{reduction}} = \frac{2NR}{3NR + R + 1}B \approx \frac{2}{3}B. \tag{S7}$$

Next, we derive the throughput of the frequency expansion scheme under anti-aliasing conditions, which is a similar process. Returning to Equation S6, we must determine the limiting factor of the term $\min(\Delta f, f_0)$. In this case, the smallest frequency spacing in the signal will be Δf_X, since $\Delta f_Y > \Delta f_X$ for this scheme. And from the anti-aliasing analysis, the lowest frequency present in the output signal will be $(r_0+1)\Delta f_Y + (1-N)\Delta f_X$. When plugging in the design choices of $\Delta f_Y = N\Delta f_X$ and $r_0 = 0$, the lowest frequency present simply becomes Δf_X. Thus, the limiting factor of the term $\min(\Delta f, f_0)$ is Δf_X for the frequency expansion scheme.

Hence, the throughput becomes $T_{\text{expansion}} = N \cdot R \cdot \Delta f_X = R\Delta f_Y$, using the design choice above for the second equality. Dividing the throughput by the available bandwidth B, we get:

$$\begin{aligned}
\frac{T_{\text{expansion}}}{B} &= \frac{R\Delta f_Y}{N\Delta f_X + (r_0+R)\Delta f_Y} \\
&= \frac{R\Delta f_Y}{N\left(\frac{1}{N}\Delta f_Y\right) + (0+R)\Delta f_Y} \\
&= \frac{R\Delta f_Y}{(1+R)\Delta f_Y} \\
&= \frac{R}{1+R} \\
&\approx 1, \quad \text{for} \quad R \gg 1.
\end{aligned}$$

Finally, the exact and approximate throughput of the frequency expansion scheme are:

$$T_{\text{expansion}} = \frac{R}{1+R}B \approx B. \tag{S8}$$

To compute the convolutional throughput a multiplicative factor of N is added to Equation S6, which results in directly applying that same factor to Equations S7 and S8. Note that in the main text, we assumed that the bandwidth B is available not only for the input and weight signals, but also for the output signal. So for the convolutional throughput, all the output spurious frequencies must fit within the bandwidth B. Fortunately, we see from Equation S4 that the highest frequency in the convolutional output is $(N-1)\Delta f_X + (r_0+R)\Delta f_Y$, which is less than B.

J. Non-WDM Throughput Derivation

Here we derive the throughput of a single fully connected layer when not using optical WDM, meaning that it is limited to the electronic components. Thus, in this case, the bandwidth B is the bandwidth available from the DPMZMs, which was already derived in the previous section. The other constraint is the photodetector with bandwidth B_{PD}. The derivation for the throughput in terms of B_{PD} is similar to that for B, except that in this case, the maximum available bandwidth is determined by the highest output neuron frequency, which yields: $B_{PD} = (r_0+R)\Delta f_Y$.

Starting with the frequency reduction scheme, we get:

$$\frac{T_{\text{reduction}}}{B_{PD}} = \frac{N\Delta f_X}{(r_0+R)\Delta f_Y}.$$

Plugging in the anti-aliasing values of $\Delta f_Y = \frac{1}{R}\Delta f_X$ and $r_0\Delta f_Y = \frac{1}{2}(N - \frac{R+1}{R})\Delta f_X$ and simplifying, we get:

$$T_{\text{reduction}} = \frac{2NR}{NR + R - 1}B_{PD} \approx 2B_{PD}, \quad \text{for} \quad N \gg 1$$

And for the frequency expansion scheme, we have:

$$\frac{T_{\text{expansion}}}{B_{PD}} = \frac{R\Delta f_Y}{(r_0 + R)\Delta f_Y}$$

Plugging in the anti-aliasing value $r_0 = 0$, we simply get:

$$T_{\text{expansion}} = B_{PD},$$

which is an exact value with no approximations.

Therefore for a given layer in the architecture, when limited to a modulation bandwidth of B and a single photodetector with bandwidth B_{PD}, the maximum throughput is:

$$T_{\text{reduction}} \approx \min(\frac{2}{3}B,\ 2B_{PD}) \tag{S9}$$

$$T_{\text{expansion}} \approx \min(B,\ B_{PD}). \tag{S10}$$

Additionally, Equations S9 and S10 show the relationship between the modulation bandwidth B (the highest frequency among the input and weight signals) and the photodetector bandwidth B_{PD} (the highest output neuron frequency). Equation S10 implies that for large R in the frequency expansion scheme, the maximum frequency contained among the input and weight signals is the same as the maximum frequency contained in the output neuron signal. This relation between B and B_{PD} can be confirmed by comparing their definitions, and supports the intuition that the electronics-limited throughput is simply the minimum of the modulator and photodetector bandwidths. And similarly for the frequency reduction scheme, Equation S9 implies that the maximum frequency among the input and weight signals is three times larger than that of the output neuron signal for large N.

K. DNN Random Partial Sum Training

Reading out the output vector after the photoelectric multiplication requires knowledge of the exact frequency content entering the system. Additionally, analog filters are required to isolate the output vector frequencies in both the frequency reduction and expansion schemes. However, in some applications it is desirable to have the flexibility to train a DNN anywhere in the spectrum without prior knowledge of the input, weight, and output frequencies.

Recall from Supplementary Section G that the spurious frequencies contain unique partial sums. Since these partial sums contain weight matrix elements, they too are trainable parameters for the DNN. Thus, assigning the output vector to random frequencies in the spectrum will result in training the DNN not with a conventional fully connected layer, but instead with a random assortment of partial sums. We call this method of DNN training *random partial sum training.*

We explored the effects of random partial sum training on larger DNNs using simulations, shown in Table S2. The hardware setup in these simulations is different than in the 3-layer experiment. In the simulations, a bandpass filter is randomly placed in the spectrum within the general range of expected frequencies for each layer.

Table S2 compares the DNN accuracy between a conventional DNN using fully connected layers with each followed by a ReLU nonlinear activation, a simulation of the MAFT-ONN with fully connected layers followed by the MZM nonlinearity, and a simulation of the MAFT-ONN with bandpass filters placed randomly in the spectrum. As shown in Table S2, the MAFT-ONN is on par with the accuracies of the conventional DNN. However, the random partial sum training version of the MAFT-ONN loses accuracy for smaller workloads, but begins to approach the performance of the conventional DNN for larger workloads. We hypothesize that the random partial sum training is less accurate because the density of partial sums in random places in the spectrum will be on average less than the density of partial sums at the output neuron values. But as the size and depth of the DNN grows, the random partial sum training seems to converge to the conventional DNN accuracy, perhaps because the expressivity of the random partial sums increases.

In our 3-layer DNN experiment, we did not filter out the spurious frequencies after the first layer and randomly chose a set of output frequencies as the one-hot vectors for the MNIST classification. Thus, we experimentally demonstrated a variant of partial sum training.

L. Encoding for Complex Matrix Products

Using complex encoding doubles the throughput of MAFT-ONN for a given amount of bandwidth B. In addition, incoming waveforms in a real-world spectral environments such as radio transmissions will not only have an arbitrary amplitude, but also an arbitrary phase for each frequency mode. Thus, we use a complex encoding scheme to reinterpret the amplitude and phase of each frequency mode into a complex number. Here we show that the matrix operations are still achieved even for complex-valued elements.

In this case, the electrical voltage signal for the inputs includes both an amplitude X_n and a phase ϕ_n^X for each frequency mode. These can be interpreted as a complex number with real part $x_n^{\text{RE}} = X_n \cos(\phi_n^X)$ and imaginary part $x_n^{\text{IM}} = -X_n \sin(\phi_n^X)$. Thus, the input activation is expressed as:

DNN Shape	Conventional Accuracy	MAFT-ONN Accuracy	MAFT-ONN Accuracy (Partial Sum Training)
$7\times 7 \to 32 \to 16 \to 10$	94.51%	93.97%	85.69%
$14{\times}14 \to 32 \to 16 \to 10$	95.12%	95.98%	86.45%
$28\times 28 \to 1000 \to 1000 \to 10$	97.98%	97.93%	87.42%
$7\times 7 \to 100 \to 100 \to 100 \to 100 \to 100 \to 10$	97.05%	95.80%	93.52%
$28\times 28 \to 100 \to 100 \to 100 \to 100 \to 100 \to 10$	97.56%	96.35%	94.85%

Table S2. A comparison of the accuracy on the MNIST dataset on conventional DNNs with fully connected layers followed by a ReLU nonlinear activation versus simulations of the MAFT-ONN with the MZM nonlinear activation.

$$V_X(t) = \sum_{n=1}^{N} X_n \cos((n_0+n)\Delta\omega_X t + \phi_n^X)$$
$$= \sum_{n=1}^{N} x_n^{\mathrm{RE}} \cos((n_0+n)\Delta\omega_X t) + x_n^{\mathrm{IM}} \sin((n_0+n)\Delta\omega_X t).$$

Similarly, each frequency mode in the weight matrix signal also has an amplitude $W_{r,n}$ and phase $\phi_{r,n}^W$, where we interpret it as a complex number with real part $w_{r,n}^{\mathrm{RE}} = W_{r,n}\cos(\phi_{r,n}^W)$ and imaginary part $w_{r,n}^{\mathrm{RE}} = -W_{r,n}\sin(\phi_{r,n}^W)$. Then the weight matrix is expressed as:

$$V_W(t) = \sum_{r=1}^{R}\sum_{n=1}^{N} W_{r,n}\cos(\omega_{r,n}^W t + \phi_{r,n}^W)$$
$$= \sum_{r=1}^{R}\sum_{n=1}^{N} w_{r,n}^{\mathrm{RE}}\cos(\omega_{r,n}^W t) + w_{r,n}^{\mathrm{IM}}\sin(\omega_{r,n}^W t).$$

Following the same procedure as in the general MAFT algorithm, multiplying these inputs and weights yields the signal of the photoelectric output vector:

$$V_Y(t) \propto \sum_{r=1}^{R} Y_r \sin(\omega_r^Y t + \phi_r^Y)$$
$$\propto \sum_{r=1}^{R} y_n^{\mathrm{RE}}\sin(\omega_r^Y t) + y_n^{\mathrm{IM}}\cos(\omega_r^Y t),$$

where $y_r^{\mathrm{RE}} = Y_r\cos(\phi_r^Y)$ and $y_r^{\mathrm{IM}} = -Y_r\sin(\phi_r^Y)$.

The expressions of y_r^{RE} and y_r^{IM} are determined by inserting the physical (amplitude and phase) representations of $V_X(t)$ and $V_W(t)$ into the general MAFT algorithm equations, then converting to the complex representation and simplifying. This yields the complex representation of the output vector:

$$y_r^{\mathrm{RE}} = \sum_{n=1}^{N} w_{r,n}^{\mathrm{RE}} x_n^{\mathrm{RE}} + w_{r,n}^{\mathrm{IM}} x_n^{\mathrm{IM}}$$
$$= \sum_{n=1}^{N} W_{r,n} X_n \cos(\phi_{r,n}^W - \phi_n^X)$$
$$y_r^{\mathrm{IM}} = \sum_{n=1}^{N} w_{r,n}^{\mathrm{RE}} x_n^{\mathrm{IM}} - w_{r,n}^{\mathrm{IM}} x_n^{\mathrm{RE}}$$
$$= \sum_{n=1}^{N} W_{r,n} X_n \sin(\phi_{r,n}^W - \phi_n^X).$$

And the physical representation of the output vector is:

$$Y_r = \sqrt{(y_r^{\mathrm{RE}})^2 + (y_r^{\mathrm{IM}})^2}$$
$$\phi_r^Y = \tan^{-1}\left(\frac{-y_r^{\mathrm{IM}}}{y_r^{\mathrm{RE}}}\right).$$

Here, we have described the physics of the MAFT-ONN when processing complex-valued signals, expressing each signal both in its physical and complex number representation. Next, we show that this physical encoding corresponds to a complex-valued matrix-vector product.

Let us define a complex-valued input vector $X^{\mathbb{C}}$ with elements $X_n^{\mathbb{C}} = x_n^{\mathrm{RE}} + i x_n^{\mathrm{IM}}$. Similarly we define a complex-valued weight matrix $W^{\mathbb{C}}$ with elements $W_{r,n}^{\mathbb{C}} = w_{r,n}^{\mathrm{RE}} + i w_{r,n}^{\mathrm{IM}}$. Finally, we define the complex-valued output vector $Y^{\mathbb{C}} = \left(W^{\mathbb{C}}\right)^* X^{\mathbb{C}}$. The elements of $Y^{\mathbb{C}}$ are:

$$
\begin{aligned}
Y_r^{\mathbb{C}} &= \sum_{n=1}^{N} \left(W_{r,n}^{\mathbb{C}}\right)^* X_n^{\mathbb{C}} \\
&= \sum_{n=1}^{N} \left(w_{r,n}^{\mathrm{RE}} + i w_{r,n}^{\mathrm{IM}}\right)^* \left(x_n^{\mathrm{RE}} + i x_n^{\mathrm{IM}}\right) \\
&= \sum_{n=1}^{N} w_{r,n}^{\mathrm{RE}} x_n^{\mathrm{RE}} + w_{r,n}^{\mathrm{IM}} x_n^{\mathrm{IM}} \\
&\quad + i \sum_{n=1}^{N} w_{r,n}^{\mathrm{RE}} x_n^{\mathrm{IM}} - w_{r,n}^{\mathrm{IM}} x_n^{\mathrm{RE}}
\end{aligned}
$$

The last expression matches that of the physical encoding, yielding $Y_r^{\mathbb{C}} = y_r^{\mathrm{RE}} + i y_r^{\mathrm{IM}}$. Therefore, the physical output of the MAFT-ONN corresponds to a complex matrix-vector product.

M. Maximizing Throughput with Scalable Multiplexing

The computational throughput discussion claimed that the bandwidth B of the throughput is not limited by the electro-optic RF components but instead by the available optical bandwidth. Hence, maximum throughput can be achieved by maximizing the use of the optical spectrum. Here we present scalable architectures that allow MAFT-ONN to increase throughput by taking advantage of optics.

Scalability in DNN Width: The "width" of a DNN refers to the number of weights that can fit into a single layer. This is directly proportional to the amount of optical bandwidth being utilizes. The two primary methods to utilize optical bandwidth with MAFT-ONN are (i) optical wavelength-division multiplexing (WDM) the frequency-encoded signals, or (ii) replacing the frequency-encoded signals with optical frequency combs. For (i), Figure S11(a) illustrates a variant of this architecture that uses optical WDM to simultaneously perform multiple matrix-vector products on each laser on the same photodetector. The incoherence between the lasers allows for each matrix-vector product to independently sum at the photovoltage output. As long as the gap between each laser wavelength is greater than the bandwidth of the photodetector, there will be no cross coupling terms between the matrix-vector products performed on each laser carrier. With the WDM version of the architecture, large matrix products can be tiled in the frequency domain, or matrix-matrix products can be frequency-multiplexed while still computing everything in a single shot. The optical bandwidth can also be used in the case of an arbitrarily deep neural network (the box labeled "Layer $j > 1$" in Figure S11(a)), where the same input vector can be independently multiplied by different weight signals for applications like convolution. There is more than 20 THz of available bandwidth among the S, C, and L telecommunication bands (1460nm-1625nm) that can be used here for optical WDM. For (ii), optical frequency combs can replace the WDM modules in Figure S11(a) for even larger throughput. Other works have experimentally demonstrated optical frequency combs with almost 1,000 THz bandwidth (59, 60). An optical AWG or waveshaper would be required to program the frequency combs.

The throughput can be further increased through spatial multiplexing with copies of the setup running in parallel, shown in Figure S11(b). Demonstrations like a photonic integrated circuit with 48 on-chip MZMs (45) show the promise for the scalability of MAFT-ONN.

Note that this throughput analysis assumes that the optical bandwidth B is the limiting factor in throughput. See Supplementary Section J for a throughput derivation that is limited to only the electronics without using optical multiplexing.

Scalability in DNN Depth: The "depth" of a DNN concerns the number of layers. Another multiplexing variant of MAFT-ONN is illustrated in Figure S11(d) that can compute an arbitrary number of DNN layers with a single set of modulators. This "loop" version uses an optical fiber delay lines as temporary optical storage to give time for the RF weights and data routing switches to operate. Just 1 km of commercially available optical fiber used as a delay line is enough to enable MHz-speed RF switches. This version of the architecture can reduce the cost, hardware complexity, and power consumption for computing DNNs with many hidden layers. The primary limiting factor here is determining how many DNN layers can be implemented before the noise degrades the performance.

N. Frequency-Domain LTI Framework

Here we show how the physics of MAFT-ONN correspond to LTI operations. Let the electrical input and LTI filter signals be $V_X(t)$ and $V_W(t)$ respectively. Assuming both signals are frequency-encoded with the same frequency spacing $\Delta\omega$ yields: $V_X(t) = \sum_{n=1}^{N} X_n \cos(n \cdot \Delta\omega \cdot t)$ and $V_W(t) = \sum_{r=1}^{R} W_r \cos(r \cdot \Delta\omega \cdot t)$.

We program the DPMZM biases such that $E_X(t)$ is SSB-SC with the right sideband suppressed and $E_W(t)$ is SSB-SC with the left sideband suppressed. Assuming both signals are frequency-encoded with the same frequency spacing $\Delta\omega$, this yields:

$$E_X(t) \propto \sum_{n=1}^{N} X_n e^{i(-n\Delta\omega + \omega_{\mathrm{LD}})t}, \tag{S11}$$

$$E_W(t) \propto \sum_{r=1}^{R} W_r e^{i(r\Delta\omega + \omega_{\mathrm{LD}})t}. \tag{S12}$$

The photoelectric multiplication then yields the output

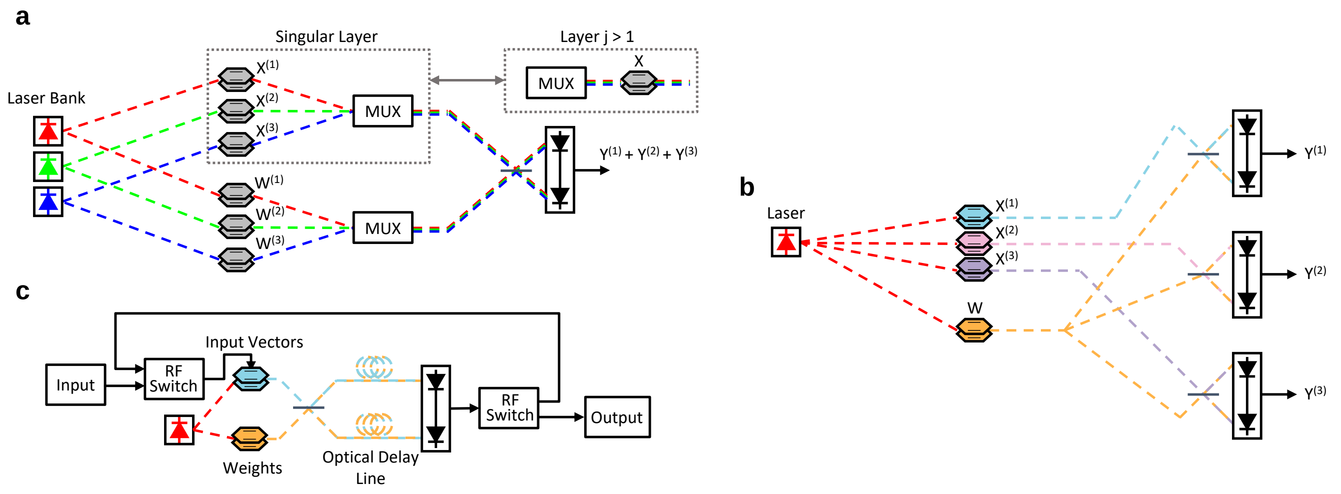

Figure S11. Architectures that exploit multiplexing to maximize the throughput for MAFT-ONN. (a) A WDM version of this architecture that expands the bandwidth-limited throughput to the wide bandwidth available in optics. Several matrix-vector products can be independently computed on different laser wavelengths, where the WDM output incoherently sums of the independent output vector signals from each laser. (b) A spatially multiplexed version of this architecture that uses optical fan-out to reuse the weight matrix for multiple input vectors. This variant can increase both throughput and energy efficiency. (c) The "loop" version of the MAFT-ONN that can implement an arbitrary number of layers with a signal set of modulators. A fiber delay line can be used to allow time for the RF weight values to update for each layer and for the RF switches to route the data.

voltage: $V_{\text{out}}(t) \propto \text{Im}\left[E_X^*(t)E_W(t)\right]$. Taking the Fourier Transform of both sides yields:

$$\mathcal{F}\left[V_{\text{out}}(t)\right] \propto \left(\tilde{E}_X \star \tilde{E}_W\right)(\omega) - \left(\tilde{E}_W \star \tilde{E}_X\right)(\omega) \quad \text{(S13)}$$

where the star indicates the cross correlation function, $\tilde{E}_X(\omega) = \mathcal{F}\left[E_X(t)\right]$ and $\tilde{E}_W(\omega) = \mathcal{F}\left[E_W(t)\right]$. The continuous cross correlation between $\tilde{E}_X(\omega)$ and $\tilde{E}_W(\omega)$ is defined as:

$$\left(\tilde{E}_X \star \tilde{E}_W\right)(\omega) = \int_{-\infty}^{\infty} \tilde{E}_X^*(\theta)\tilde{E}_W(\omega+\theta)\ d\theta \quad \text{(S14)}$$

Equations S11 through S14 can be combined and simplified to yield:

$$\mathcal{F}\left[V_{\text{out}}(t)\right] \propto \sum_{r=1}^{R}\sum_{n=1}^{N} X_n^* W_x \delta(\omega-(x+n)\Delta\omega) - X_n W_x^* \delta(\omega+(x+n)\Delta\omega). \quad \text{(S15)}$$

where $\delta(\cdot)$ is the Dirac delta function.

Note that instead of the frequencies subtracting like in Supplementary Section G, they add here when the DP-MZM biases are programmed to place $E_X(t)$ and $E_W(t)$ on opposite sidebands. The terms in the summation containing $X_n^* W_x$ strictly correspond to the positive frequencies and the terms with $X_n W_x^*$ strictly correspond to the negative frequencies. Hence there is no aliasing between the two terms in the summation. And when taking the Inverse Fourier Transform of Equation S15, a summation of real-valued sine waves with various phases emerges.

Due to the lack of positive/negative frequency aliasing between the terms in Equation S15 we can just consider the terms that correspond to the positive frequencies for the LTI analysis:

$$\mathcal{F}\left[V_{\text{out}}(t)\right]_+ \propto \sum_{r=1}^{R}\sum_{n=1}^{N} X_n^* W_x \delta(\omega-(x+n)\Delta\omega). \quad \text{(S16)}$$

Now we define the frequency-domain LTI signals and show that the LTI convolution operator matches the physics of MAFT-ONN. We first define a simple LTI scheme were we allow information to be encoded at DC. Let $x[n] \equiv X_n \rightarrow n\Delta\omega$ and $w[n] \equiv W_n \rightarrow n\Delta\omega$. Hence the values of $x[n]$ and $w[n]$ are mapped to the frequency mode at $n\Delta\omega$. Thus the LTI signals are:

$$x[n] = \sum_{m=1}^{N} X_m \delta[n-m]$$

$$w[n] = \sum_{m'=1}^{R} W_{m'} \delta[n-m'].$$

where $\delta[\cdot]$ is the Kronecker delta function and m' is distinguished from m for indexing convenience.

Then the convolution operator $(x * w)[n]$ yields:

$$\begin{aligned}(x * w)[n] &= \sum_{k=-\infty}^{\infty} x[k]w[n-k] \\ &= \sum_{m'=1}^{R}\sum_{m=1}^{N} X_m W_{m'} \delta[n-(m'+m)]. \quad \text{(S17)}\end{aligned}$$

Therefore when considering real values for $x[n]$ and $w[n]$, the LTI convolution in Equation S17 matches the MAFT-ONN physics in Equation S16. When considering complex values we still achieve a useful LTI-like convolution.

However, this LTI scheme requires programming the information of $x[0]$ and $w[0]$ at DC. To avoid this we may offset the indexing by 1 such that $x[n] \equiv X_{n+1} \rightarrow (n+1)\Delta\omega$ and $w[n] \equiv W_{n+1} \rightarrow (n+1)\Delta\omega$. Now the values $x[n]$ and $w[n]$ correspond to the frequency mode at $(n+1)\Delta\omega$. The new LTI signals are:

$$x[n] = \sum_{m=1}^{N} X_m \delta[n+1-m]$$

$$w[n] = \sum_{m'=1}^{R} W_{m'} \delta[n+1-m'].$$

And now the convolution operator $(x * w)[n]$ yields:

$$\begin{aligned}(x * w)[n] &= \sum_{k=-\infty}^{\infty} x[k]w[n-k] \\ &= \sum_{m'=1}^{R}\sum_{m=1}^{N} X_m W_{m'} \delta[n+2-(m'+m)]. \end{aligned} \quad \text{(S18)}$$

With this framework, the output LTI signal $y[n] = (x * w)[n]$ can be physically interpreted similarly as $x[n]$ and $w[n]$ where the value of $y[n]$ corresponds to the frequency mode at $(n+1)\Delta\omega$. This keeps the frequency-domain interpretation of $x[n]$, $w[n]$, and $y[n]$ consistent while avoiding programming information at DC.

However, note that with this offset LTI framework, comparing Equation S18 with the physics of MAFT-ONN in Equation S16 reveals that MAFT-ONN actually physically computes: $y_{\text{MAFT}}[n] = y[n-1] = (x*w)[n]*\delta[n-1]$. This means that MAFT-ONN physically pushes $y[n]$ up by one $\Delta\omega$ for every LTI convolution. For the overwhelming majority of use-cases this is non-problematic as long as one properly keeps track of the frequencies. But if one truly wishes, the extra $\delta[n-1]$ can be removed with another layer of MAFT by multiplying $y_{\text{MAFT}}[n]$ with a single tone using same-sideband DPMZM biasing. Because LTI operations are cascadable and the order does not matter, this can be done at the very end of a chain of MAFT-ONN LTI convolutions.

Furthermore, the *linearity* and *time invariance* requirements of this frequency-domain LTI framework are clearly upheld by the physics of MAFT-ONN in Equation S16, which has been validated by the numerous experiments in this work. The MAFT-ONN linearity is upheld as long as the DPMZMs operate in their linear regime and the time invariance is upheld from the fact that the same LTI convolution is computed regardless of where in the spectrum the signal lies. Due to how we mapped the LTI signals to the physics, all signals anywhere in the MAFT-ONN frequency domain LTI framework will have zero magnitude for $n < 0$.

Note that the Dirac delta functions in Equation S16 can be pulse-shaped using a frequency communications scheme like orthogonal frequency division multiplexing (OFDM) and still essentially retain the LTI functionality albeit with finite resolution.

O. Communication Link Gain Analysis

The gain of single layer of MAFT-ONN is expressed below (61):

$$g\ (\text{linear}) = \frac{\pi^2}{8}\left(\frac{R_{PD}\gamma P_{\text{LD}}}{V_\pi}\right)^2 R_i R_o \left|H_{PD}(f)\right|^2 \left\langle V_W^2(t)\right\rangle \quad \text{(S19)}$$

where R_{PD} is the responsivity of the photodetector, γ is the gain of the optical link (modulator insertion loss, fiber propagation loss, optical amplifiers, etc.), P_{LD} is the laser power, V_π is the voltage required to reach π phase shift on the modulators, R_i and R_o are the input and output resistances respectively, $H_{PD}(f)$ is the frequency response of the photodetector, and $\left\langle V_W^2(t)\right\rangle$ is the time-averaged power of the weight matrix signal. Note that this equation is for a receiverless link (no RF amplifier following the balanced photodetector).

Figure S12 illustrates a trade-space between the laser power, the weight signal power, and an RF amplifier. In the plot, V_π is 6 V,R_{PD} is 1 A/W, γ is -6 dB, R_i and R_o are 50 Ω, and H_{PD} is 1/2. Since the power of the weight signal can be adjusted to fit within the linear regime of any modulator, the gain curves are independent of the V_π of the modulators and instead depend on $\left\langle V_W^2(t)\right\rangle$. However, the V_π will still determine the threshold of nonlinear regime of the modulator implementing the nonlinear activation.

In our experiment, we found that our DPMZM with $V_\pi \approx 6\ V$ did not exhibit nonlinear behavior until the input signal reached around $P_{\text{nonlin}} = V_\pi^2/R_i \approx 27$ dBm. To alleviate the requirement of high power signals to reach the nonlinear regime, note that in principle, modulators with $V_\pi \approx 1$ mV can be fabricated (62). In that case, the nonlinear power threshold of the modulator is $P_{\text{nonlin}} \approx -47$ dBm. This even allows for input RF signals with -85 dBm of power, which is typically con-

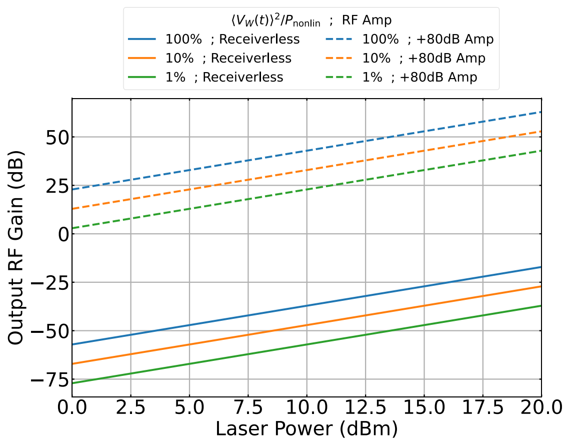

Figure S12. A communication link gain analysis, illustrating the trade-space between the laser power, weight signal power, and RF amplifier gain from Equation S19. The weight signal can be any power as long as it stays within the linear regime of the modulator, where nonlinear power threshold of the modulator is P_{nonlin}.

sidered the minimum usable power level for communications (63), to be amplified enough to reach the nonlinear regime. Therefore in some scenarios, the gain from the laser may allow for receiverless operation, and in others an amplifier either before or after the modulators may be required.

P. Latency Estimation Model

Here we estimate the theoretical fastest latency of various digital processors for the task of applying a filter to an incoming wireless signal with bandwidth B. The filter is a simple 1-dimensional convolution in the frequency-domain with frequency resolution Δf. Hence for a signal with bandwidth B and frequency resolution Δf, the Nyquist sampling theorem requires at least $N = 2\lceil \frac{B}{\Delta f} \rceil$ points to digitally capture this signal. Finally, the filtered signal is moved to a device that can implement the next stage of processing as necessary.

Note that these are models of electronics working at their idealized peak speeds, using optimistic parameters (compared to what is used in practice), with 100% processor and memory utilization, without the additional processing management overhead, assuming no data rate degradation from high clock speeds, and without throttling due to overheating. Hence, we are comparing MAFT to the theoretical limits of electronics that are usually not even achieved in practice.

Figure 4A in the main text illustrates the various components modeled here that contribute to the latency of each computing architecture. Note that GPUs will likely suffer the most additional overhead that cannot be deterministically predicted by a model, which influences the latencies achieved in practice.

GPU Latency: The latency of filtering the RF signal using a typical GPU system is estimated to be:

$$\begin{aligned}\tau_{\text{GPU}} = \tau_{\text{Gabor}} + \tau_{\text{JESD}} + \tau_{\text{PCIe}} + \tau_{\text{VRAM}} \\ + \tau_{\text{MAC}} + \tau_{\text{VRAM}}\end{aligned}$$

The first term τ_{Gabor} is the Gabor limit, which is a fundamental constraint between time and frequency resolution. In order to achieve frequency resolution Δf, the signal must be sampled for $\tau_{\text{Gabor}} = 1/\Delta f$ seconds. We assume that the physical latency of the ADC is negligible compared to the Gabor limit. For simplicity we assume that the ADC sampling rate is exactly double the RF bandwidth, thus sampling at a rate of 2B samples/second to produce 2N samples. (The factor of two on the number of points derives from sampling both the I and Q components of the RF signal.) To trade off between speed and accuracy, we assume each sample has 16-bit precision ($b = 2$ bytes), thus producing $D = 2Nb$ bytes of data.

The second term τ_{JESD} is the latency due to data transfer from the ADC to the CPU. Here we assume the data rate is the maximum possible $r_{\text{JESDC}} = 32$ Gb/s $= 4$ GB/s for JESD204C and $J_{\text{lanes}} = 16$ parallel lanes. Hence, this latency is $tau_{\text{JESD}} = \frac{D}{J_{\text{lanes}} r_{\text{JESDC}}}$.

The third term τ_{PCIe} is the latency due to data transfer to and from the CPU to the GPU, typically via PCIe. Here we assume the maximum possible data rate of PCIe 4.0 of $r_{\text{PCIe4r}} = 2$ GB/s with $P_{\text{lanes}} = 16$ lanes to yield a latency of $\tau_{\text{PCIe}} = \frac{D}{P_{\text{lanes}} r_{\text{PCIe4}}}$.

The fourth and sixth terms τ_{VRAM} is the time it takes for the GPU to transfer data from its VRAM to its compute. This term depends on the specific memory bandwidth of the GPU r_{VRAM}, and is thus $\tau_{\text{VRAM}} = \frac{D}{r_{\text{VRAM}}}$.

The fifth term τ_{MAC} is the time it takes to compute the RF filter. This is approximated by the expression: $\tau_{\text{MAC}} = \frac{O(N)}{r_{\text{clock}} n_{\text{PE}}} = \frac{2N}{r_{\text{clock}} n_{\text{PE}}}$, where $O(N)$ is the number of MACs for the computation, the factor of 2 comes from the fact that the RF signal is complex, r_{clock} is the clock speed of the GPU and n_{PE} is the number of parallel processing cores. The clock speed and number of processing cores depends on the GPU.

FPGA Latency: For digital processors, there is a trade-off between the size of the RAM and the amount of overhead for data management. FPGAs have significantly less RAM than GPUs but in return can directly process specialized operations with minimal overhead. The estimated ideal latency of an equivalent FPGA RF filter is:

$$\begin{aligned}\tau_{\text{FPGA}} = \tau_{\text{Gabor}} + \tau_{\text{JESD}} + \tau_{\text{RAM}} + \tau_{\text{PL mem}} \\ + \tau_{\text{MAC}} + \tau_{\text{RAM}}\end{aligned}$$

The terms τ_{Gabor} and τ_{JESD} are the same as in the GPU model.

GPU Model	$r_{\mathbf{clock}}$	$n_{\mathbf{PE}}$	$r_{\mathbf{VRAM}}$
GeForce RTX 4090	2253 MHz (2520 MHz boosted)	16,384 CUDA cores	1,008 GB/s
RTX 5000 Ada Generation	1155 MHz (2550 MHz boosted)	12,800 CUDA cores	576 GB/s

Table S3. The parameters and devices used in the latency estimation model for the GPU RF receiver architecture. All our GPU latency estimations use the boosted clock frequencies.

FPGA Model	$n_{\mathbf{PE}}$	$r_{\mathbf{RAM}}$	$M_{\mathbf{BRAM}}$	$M_{\mathbf{URAM}}$
Versal AI Edge VE2802	1312 DSP cores	102.4 GB/s	21.1 Mb	74.3 Mb
Versal HBM Series VH1782	10848 DSP cores	819.2 GB/s	132 Mb	541 Mb

Table S4. The parameters and devices used in the latency estimation model for the FPGA RF receiver architecture. Note that we assume $r_{\mathrm{clock}} = 500$ MHz for our FPGA estimations.

The third and seventh term τ_{RAM} is the latency due to data movement to/from the DRAM/HBM and the BRAM/URAM. This is dominated by the maximum theoretical DRAM/HBM rate r_{RAM} and the number of buses to the DRAM/HBM R_{lanes}, both of which depend on the specific FPGA model and setup. The term τ_{read} accounts for the latency of initializing each memory read. (Thus, we capture the penalty for having small programmable logic (PL) RAM. Otherwise this model would not distinguish between an FPGA with a kilobyte versus a gigabyte of PL RAM.) In memory architectures like DDR4, the latency for each memory call is dominated by the CAS latency, which is usually given in units of number of clock cycles. Because there are various types of memory architectures we simplify the analysis by estimating the optimal read latency n_{read} as 10 cycles for DRAM and HBM and 2 cycles for BRAM and URAM. Thus: $\tau_{\mathrm{read}} = \lceil \frac{D}{M} \rceil \frac{n_{\mathrm{read}}}{r_{\mathrm{clock}}}$ where M is the total PL RAM available to the FPGA and r_{clock} is the clock speed of the FPGA (for simplicity we assume the clock shared for the DSP blocks and the RAM). Therefore: $\tau_{\mathrm{RAM}} = \frac{D}{R_{\mathrm{lanes}} r_{\mathrm{RAM}}}$.

The fourth and sixth term $\tau_{\mathrm{PL\ mem}}$ is the latency for transferring data to/from the PL memory (including BRAM and URAM) to the DSP blocks. For simplicity we assume the bottleneck here is which is determined by minimum of the total BRAM and URAM memory bandwidths, r_{BRAM} and r_{URAM}. Because FPGA spec sheets usually only specify the amount of BRAM and URAM in Megabytes, we can estimate the memory bandwidth by using the basic architecture of each RAM. Each BRAM block contains 36 kb with 2 36 bit ports, and each URAM block contains 288 kb with 2 72 bit ports. Hence for a given amount of BRAM memory m_{BRAM} and URAM memory M_{URAM}, the memory bandwidths can be estimated as $r_{\mathrm{BRAM}} = 2 \cdot 36\text{bits} \cdot \lceil \frac{M_{\mathrm{BRAM}}}{36\text{kb}} \rceil r_{\mathrm{clock}}$ and $r_{\mathrm{URAM}} = 2 \cdot 72\text{bits} \cdot \lceil \frac{M_{\mathrm{URAM}}}{288\text{kb}} \rceil r_{\mathrm{clock}}$.

Therefore: $\tau_{\mathrm{PL\ mem}} = \frac{1}{2}\left(1/r_{\mathrm{BRAM}} + 1/r_{\mathrm{URAM}}\right) D + \tau_{\mathrm{read}}$. Note that for simplicity we assume an even split in using BRAM and URAM, and the τ_{read} here depends on the read latency of BRAM/URAM instead of DRAM/HBM.

The term τ_{MAC} is the computational time it takes for the DSP blocks to compute the filter. Similarly to the GPU case, this is estimated as: $\tau_{\mathrm{MAC}} = \frac{O(N)}{r_{\mathrm{clock}} n_{\mathrm{DSP}}} = \frac{2N}{r_{\mathrm{clock}} n_{\mathrm{DSP}}}$, where r_{clock} is the FPGA clock frequency and n_{DSP} is the number of DSP blocks on the FPGA, which depends on the model.

ADC-Integrated RF SoC Latency: Finally, the highest-performing ultra low-latency RF signal processing architecture are FPGAs with integrated ADCs and RF SoCs. This eliminates the bottleneck of the data transfer between the ADC and the FPGA PL memory by directly linking them together. The estimated ideal latency of this architecture is:

$$\tau_{\mathrm{RFSoC}} = \tau_{\mathrm{Gabor}} + \tau_{\mathrm{PL\ mem}} + \tau_{\mathrm{RAM}}$$

All of the terms here have already been defined in the previous sections.

MAFT Latency: The latency of MAFT with J cascaded layers is:

$$\tau_{\mathrm{MAFT}} = J(\tau_{MZM} + \tau_{PD} + \tau_{RF} + \tau_{\mathrm{prop}}) + \tau_{\mathrm{Gabor}},$$

where τ_{MZM} is the reciprocal of the bandwidth of the MZM, τ_{PD} is the reciprocal of the bandwidth of the photodetector, τ_{RF} is the combined delay due to the bandwidth of additional RF components like a bandpass filter or amplifier, and τ_{prop} is the data movement in the form of propagation of the frequency-encoded electromagnetic waves.

The value of τ_{MZM} highly depends on the material used for the MZM. Very high speed commercial MZMs

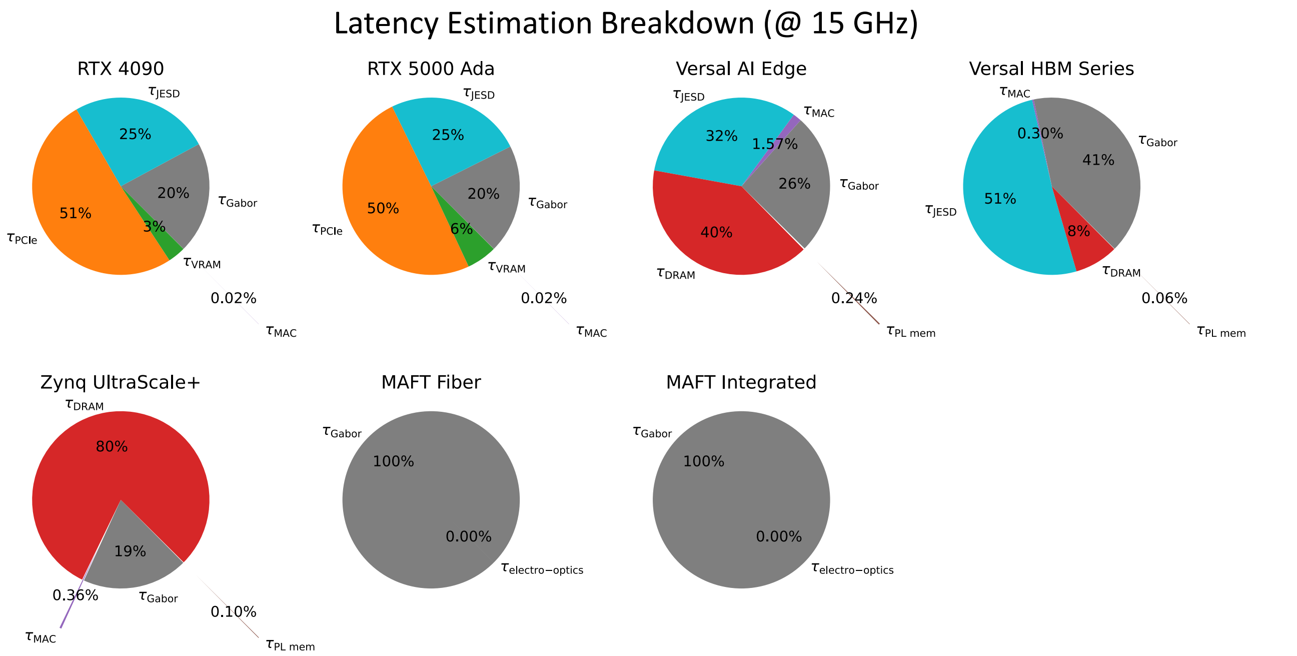

Figure S13. A breakdown of the contributions to the total latency for the different evaluated RF receiver architectures evaluated at $B = 15$ GHz of input RF bandwidth. The breakdown varies depending on B, where for lower values of B the Gabor limit dominates and for higher values of B the data movement dominates.

**RF SoC Model**	$n_{\mathbf{PE}}$	$r_{\mathbf{RAM}}$	$M_{\mathbf{BRAM}}$	$M_{\mathbf{URAM}}$
Zynq UltraScale+ RFSoC ZU49DR	4272 DSP cores	19.2 GB/s (DDR4 64-bit)	38.0 Mb	22.5 Mb

Table S5. The parameters and devices used in the latency estimation model for the ADC-integrated FPGA RF SoC receiver architecture. Note that we assume $r_{\text{clock}} = 500$ MHz for these latency estimations.

typically have up to 40 GHz bandwidth, contributing ~ 25 ps delay. The photodetector latency can be separated into the RC time constant and carrier transit time: $\tau_{PD} = \sqrt{\tau_{RC}^2 + \tau_{transit}^2}$ (64). Whether the RC or carrier transit time will dominate the latency highly depends on the photodetector design. State-of-the-art commercial photodetectors have up to 100 GHz bandwidth, thus contributing ~ 10 ps latency. The value of τ_{RF} is variable and will depend on the use-case; in some scenarios the RF bandpass filter and amplifier are optional. If using a narrow-band RF filter to remove spurious frequencies, then τ_{RF} will strongly dominate the physical latency. Thus, one benefit of keeping the spurious frequencies is to reduce the latency. Finally, the propagation time τ_{prop} is determined by the length of the optical and electrical paths. The frequency-encoded electromagnetic waves will pass through these paths at approximately the speed of light, depending on the refractive index and waveguide properties. The combined length of commercial fiber-optical components typically have tens of centimeters of optical path length after trimming the fiber leads, contributing ~ 300 ps of latency. The electrical RF connections will contribute a similar latency. The optical path length can be shortened to tens of millimeters by implementing this architecture on a photonic integrated circuit (45), reducing the latency to ~ 30 ps. Therefore depending on the scenario, the latency of this architecture will be dominated by data movement at the speed of light, τ_{prop}.

In our experiment, we measured a time-of-flight latency of 60 ns per layer using DPMZMs with 25 GHz bandwidth, a balanced photodetector with 45 MHz bandwidth, and an RF amplifier with 1 GHz bandwidth. In addition, our experimental setup contains approximately 10 meters of signal propagation, given that standard commercial fiber components have 1 meter fiber leads on each side, plus the length of the RF coaxial cables. Thus, in our experiment, the dominant sources of time-of-flight latency are $\tau_{PD} \approx 1/45$ MHz ≈ 25 ns and $\tau_{\text{prop}} \approx \frac{10 \text{ m}}{3 \cdot 10^8 \text{m/s}} \approx 35$ ns.

For the RF latency analysis in main text Figure 4C, because no electronic RF filters are required to implement a simple frequency-domain operation with MAFT, $\tau_{\text{RF}} = 0$. The final term τ_{Gabor} is included so that the information from the MAFT-filtered signal can be extracted by the decision system. The latency contribution

MAFT-ONN Variant	τ_{MZM}	τ_{prop}	τ_{PD}
MAFT (Untrimmed fiber)	25 ps	35 ns	10 ps
MAFT (Integrated)	25 ps	30 ps	10 ps

Table S6. The parameters used in the latency estimation model for the MAFT variants of the RF receiver architecture.

from τ_{Gabor} will be dominated by the Gabor limit regardless of whether an ADC or analog filter bank is used to read out the output signal.

Gabor Limit: The fundamental limit to the latency of digitally reading out the signal is the amount of time it takes to read out the frequency mode: $\tau_{\text{Gabor}} = 1/\Delta f$.

Latency Specs: This latency estimation model takes common specifications from data sheets to estimate the latency. Tables S3 to S6 show the various parameters used to create the latency comparison plot in main text Figure 4C.

Latency Breakdown: Figure S13 shows how much each variable contributes to the total latency of each evaluated RF receiver architecture. As is already well-known, this illustrates that the primary bottleneck in electronic processing latency is data movement. Hence, the Shannon capacity-limited data movement to compute is what gives MAFT the advantage in latency compared to electronic versions.

Q. Compute Density Analysis

The maximum number of neurons (frequency modes) that fits on a single electro-optical modulator determines the density of compute achievable by MAFT-ONN, which influences its scalability. The maximum number of frequency modes that a single modulator can support is limited by either the bandwidth supported by the modulator (depending on the frequency spacing) or the maximum non-destructive power the modulator can tolerate. For this analysis, let the input RF signal $V(t)$ have N frequency modes at $f_n = (n_0 + n)\Delta f$, where $n \in [1, N]$. Then the highest RF frequency is $(n_0 + N)\Delta f$.

For the former limit, let the maximum bandwidth of an electro-optical modulator be B (assuming all other components also support that bandwidth). Then the following inequality applies: $n_0 + N\Delta f \leq B$. This is the *bandwidth limit* on the number of neurons that can fit on a single modulator.

For the latter limit, let the maximum non-destructive power of the modulator be P_{mod} and the input resistance to the modulator be R_i. This means that the average power of $V(t)$ must be lower than P_{mod}, yielding $\langle V^2(t) \rangle \leq P_{\text{mod}}$. For simplicity, let each of the N frequency-mode neurons in $V(t)$ have the same amplitude V_0. Then:

$$\langle V^2(t) \rangle = N \frac{V_0^2}{2R_i}$$

where the factor of 1/2 comes from using the RMS voltage to calculate the average power.

Here, we begin to observe the nature of this limit. When $\langle V^2(t) \rangle = P_{\text{mod}}$, the power of each individual frequency-mode neuron is $P_{\text{neuron}} = \frac{V_0^2}{2R_i} = \frac{P_{\text{mod}}}{N}$. That is, because of the non-destructive power limit of the modulator, the total power of $V(t)$ must be distributed between the neurons. When N becomes too large, the power of each frequency mode will eventually become so small that they are covered by the noise floor. This is the *noise limit* on the number of neurons a single modulator can support. Hence, the larger N is per modulator, the worse the SNR and bit-precision of the individual neurons. The noise power spectral density of an IMDD link is (61):

$$\mathcal{N} = gk_BT + k_BT + 2q\left(\frac{R_{\text{PD}}\gamma P_{\text{LD}}}{2}\right) R_o \left|H_{PD}(f)\right|^2 + \text{RIN} \cdot \left(\frac{R_{\text{PD}}\gamma P_{\text{LD}}}{2}\right)^2 R_o \left|H_{PD}(f)\right|^2$$

where g is the gain of the IMDD link defined in Supplementary Section O, k_B is Boltzmann's constant, T is the temperature of the system, q is the elementary charge constant, RIN is the relative intensity noise (i.e. laser noise, optical amplifier noise), and the other variables are defined in Supplementary Section O. The first term is the input thermal noise multiplied by the gain of the IMDD link, the second term is the thermal noise of the output of the IMDD link, the third term is shot noise, and the fourth term is from miscellaneous sources of noise.

Given that $V(t)$ occupies bandwidth $N\Delta f$, the noise power after the IMDD link is $P_{\text{IMDD}} = N\Delta f\mathcal{N}$. Hence, the SNR of each individual frequency-mode neuron after the IMDD link (assuming no MAFT operations are computed on it, for simplicity) is $gP_{\text{neuron}}/P_{\text{IMDD}}$. The fundamental limit is when the SNR is 1, yielding:

$$\text{SNR}_{\text{neuron}} = \frac{gP_{\text{neuron}}}{P_{\text{IMDD}}} = \frac{gP_{\text{mod}}}{N^2\Delta f\mathcal{N}} \geq 1$$

$$N \leq \sqrt{\frac{gP_{\text{mod}}}{\Delta f\mathcal{N}}}$$

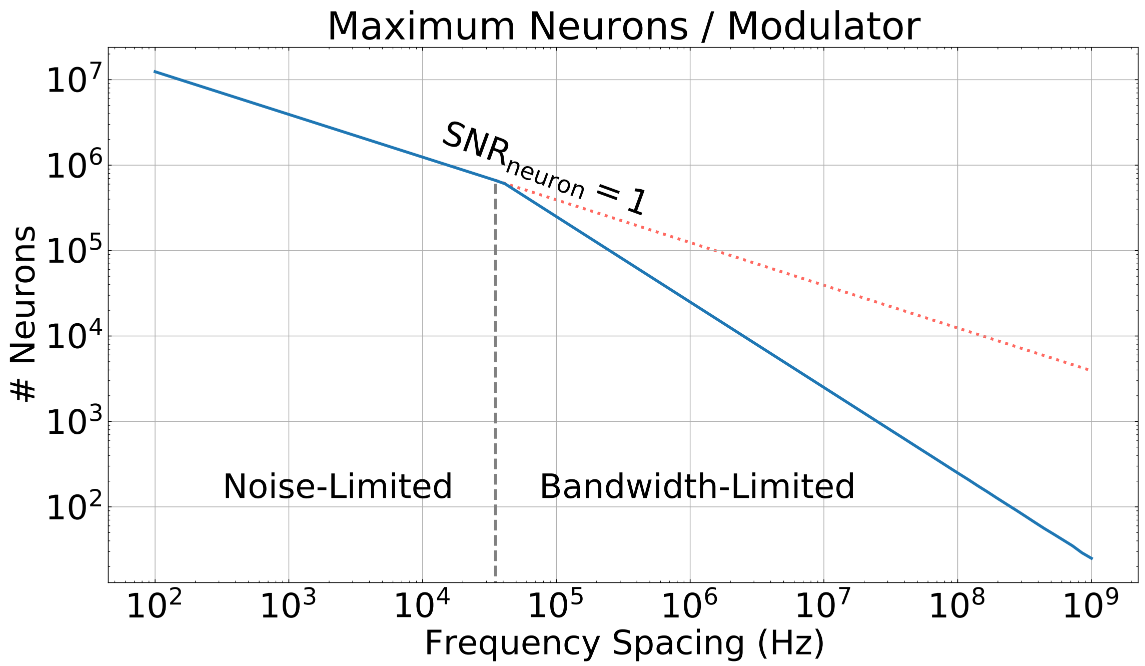

Figure S14. The limit on the number of frequency-mode neurons a single electro-optical modulator can support. For large frequency spacing, the limit is the bandwidth of the modulator, as the signal will occupy more bandwidth. For smaller frequency spacing, the limit is determined by the maximum non-destructive power of the modulator and the noise of the optical components. This is because the power per neuron frequency mode decreases as the number of neurons increases, eventually reducing the SNR of each neuron to 1. Note for this figure, the gain g is -60 dBm, showing that even with small IMDD link gain a single modulator can potentially simultaneously support millions of neurons.

Combining the bandwidth limit with the noise limit, the number of neurons that can be supported on a single modulator is:

$$N \leq \min\left(\sqrt{\frac{gP_{\text{mod}}}{\Delta f \mathcal{N}}}, \frac{B - n_0}{\Delta f}\right)$$

Figure S14 shows the relationship between the maximum number of neurons and maximum frequency spacing, with $n_0 = 0$ to maximize the number of neurons, $T = 290$ K at the standard temperature, $P_{\text{LD}} = 15$ dBm (approximately the same as our laser), $P_{\text{mod}} = 28$ dBm (approximately the same as our Exail DPMZM), $B = 25$ GHz (approximately the same as our Exail DPMZM), $\langle V_W^2(t)\rangle / P_{\text{nonlin}} = 50\%$ (see Figure S12) and RIN $= 0$. For this analysis we exclude the contribution of RIN because common-mode sources of noise are cancelled out at the balanced photodetector. The line for the noise limit is where the neuron SNR is 1, and as previously calculated the neuron SNR (and thus bit precision) increases when moving away from the noise limit. This is consistent with the decreasing bit precision in Figure 2B for the scalar-scalar to matrix-vector characterizations.

When scaling MAFT-ONN, the bit precision is maximized when minimizing the number of neurons per modulator through the use of the optical multiplexing methods described in Supplementary Section M. Thus, the tradeoff between frequency spacing, compute density, and bit precision can be taken into account for any use-case.

As shown in Figure S14, the scalability benefit of MAFT-ONN is that it enables computational capabilities with high density as experimentally demonstrated in the main text. Table S7 compares these characteristics with experimental demonstrations of state-of-the-art ONNs. The theme is that there is a distinction between partial sum multiplication (physically multiplying each input neuron with each weight element) and the summation of those partial elements to complete a full matrix multiplication. For example, (33) uses diffractive optics for the partial sum accumulation and (31) uses a series of photodetectors, but each approach still requires a separate electro-optical element to physically implement the element-wise multiplications between the input and weight partial sum elements. This limits the number of parameters that can be used to perform experimental inference, because increasing the size of the DNN entails fabricating a greater number of electro-optical devices for each neuron.

Additionally, for the nonlinear activation each architecture requires another separate electro-optical device

Ref	Matrix Element Partial-Sum Multiplication (excluding summation)	In-line Nonlinear Activation	# Output Neurons / Spatial Mode / Time Step (nonlinear activation)	Programmable Arbitrary Matrix Multiplication	Number of Weight Parameters (Optical)
Shen 2017 (11)	MZI Mesh	No	N/A	No (Unitary)	16
Xu 2021 (20)	MZM modulation	No	N/A	Yes	795
Feldmann 2021 (21)	Optical attenuation	No	N/A	Yes	16
Zhu 2022 (33)	Complex MZI modulation	Yes	1	No (Hadamard)	170
Chen 2023 (32)	Homodyne detection	Yes	1	No (Nonlinear)	79,410
Ashtiani 2022 (31)	Optical attenuation	Yes	1	Yes	66
This work	Photoelectric multiplication	Yes	39,100	Yes	20,600

Table S7. Comparison table evaluating the experimental demonstrations of state-of-the-art ONNs.

for each individual output neuron. These limitations restrict the practicality of these architectures to compute practical deep learning tasks (hundreds of millions of MACs per inference) when thousands of individual electro-optical elements must be fabricated. MAFT-ONN improves upon these architectures by performing both all the partial-sum multiplications and the partial-sum accumulation in a single shot with a single device. Hence, MAFT-ONN demonstrates highly efficient use of individual electro-optic devices, totaling only 4 DPMZMs and 2 photodetectors for the entire experiment.

REFERENCES AND NOTES

1. J. Gao, D. Wu, F. Yin, Q. Kong, L. Xu, S. Cui, Metaloc: Learning to learn wireless localization. *IEEE J. Sel. Areas in Commun.* **41**, 3831–3847 (2023).

2. L. Jiao, Y. Shao, L. Sun, F. Liu, S. Yang, W. Ma, L. Li, X. Liu, B. Hou, X. Zhang, R. Shang, Y. Li, S. Wang, X. Tang, Y. Guo, Advanced deep learning models for 6G: Overview, opportunities and challenges. *IEEE Access* **12**, 133245–133314 (2024).

3. J. M. de la Rosa, AI-managed cognitive radio digitizers. *IEEE Circ. Syst. Mag.* **22**, 10–39 (2022).

4. G. C. Lee, A. Weiss, A. Lancho, J. Tang, Y. Bu, Y. Polyanskiy, G. W. Wornell. “Exploiting temporal structures of cyclostationary signals for data-driven single-channel source separation,” in *2022 IEEE 32nd International Workshop on Machine Learning for Signal Processing (MLSP)* (IEEE, 2022), pp. 1–6.

5. K. Youssef, L. Bouchard, K. Haigh, J. Silovsky, B. Thapa, C. V. Valk, Machine learning approach to rf transmitter identification. *IEEE J. Radio Freq. Identif.* **2**, 197–205 (2018).

6. G. Shen, J. Zhang, A. Marshall, L. Peng, X. Wang, “Radio frequency fingerprint identification for lora using spectrogram and CNN,” in *IEEE INFOCOM 2021-IEEE Conference on Computer Communications* (IEEE, 2021), pp. 1–10.

7. J. Ren, X. Jiang, Regularized 2-D complex-log spectral analysis and subspace reliability analysis of micro-doppler signature for uav detection. *Pattern Recognit.* **69**, 225–237 (2017).

8. H.-T. Peng, J. C. Lederman, L. Xu, T. F. de Lima, C. Huang, B. J. Shastri, D. Rosenbluth, P. R. Prucnal, A photonics-inspired compact network: Toward real-time AI processing in communication systems. *IEEE J. Sel. Top. Quantum Electron.* **28**, 1–17 (2022).

9. L. J. Wong, W. H. Clark, B. Flowers, R. M. Buehrer, W. C. Headley, A. J. Michaels, An RFML ecosystem: Considerations for the application of deep learning to spectrum situational awareness. *IEEE Open J. Commun. Soc.* **2**, 2243–2264 (2021).

10. N. Piovesan, D. López-Pérez, A. De Domenico, X. Geng, H. Bao, M. Debbah, Machine learning and analytical power consumption models for 5g base stations. *IEEE Commun Mag* **60**, 56–62 (2022).

11. Y. Shen, N. C. Harris, S. Skirlo, M. Prabhu, T. Baehr-Jones, M. Hochberg, X. Sun, S. Zhao, H. Larochelle, D. Englund, M. Soljačić, Deep learning with coherent nanophotonic circuits. *Nat. Photon.* **11**, 441–446 (2017).

12. H. Zhang, M. Gu, X. D. Jiang, J. Thompson, H. Cai, S. Paesani, R. Santagati, A. Laing, Y. Zhang, M. H. Yung, Y. Z. Shi, F. K. Muhammad, G. Q. Lo, X. S. Luo, B. Dong, D. L. Kwong, L. C. Kwek, A. Q. Liu, An optical neural chip for implementing complex-valued neural network. *Nat. Commun.* **12**, 457 (2021).

13. H. Bagherian, S. Skirlo, Y. Shen, H. Meng, V. Ceperic, M. Soljacic, On-chip optical convolutional neural networks, arXiv:1808.03303 [cs.ET] (2018).

14. S. Bandyopadhyay, A. Sludds, S. Krastanov, R. Hamerly, N. Harris, D. Bunandar,M. Streshinsky, M. Hochberg, D. Englund, Single chip photonic deep neural network with accelerated training, arXiv:2208.01623 [cs.ET] (2022).

15. S. Kovaios, I. Roumpos, M. Moralis-Pegios, G. Giamougiannis, M. Berciano, F. Ferraro, D. Bode, S. A. Srinivasan, M. Pantouvaki, N. Pleros, A. Tsakyridis, Scaling photonic neural networks: A silicon photonic gemm leveraging a time-space multiplexed xbar. *J. Lightwave Technol.* **42**, 7825–7833 (2024).

16. S. Xu, J. Wang, W. Zou, Optical convolutional neural network with WDM-based optical patching and microring weighting banks. *IEEE Photonics Technol. Lett.* **33**, 89–92 (2021).

17. A. N. Tait, T. F. de Lima, E. Zhou, A. X. Wu, M. A. Nahmias, B. J. Shastri, P. R. Prucnal, Neuromorphic photonic networks using silicon photonic weight banks. *Sci. Rep.* **7**, 7430 (2017).

18. J. Feldmann, N. Youngblood, C. D. Wright, H. Bhaskaran, W. H. Pernice, All-optical spiking neurosynaptic networks with self-learning capabilities. *Nature* **569**, 208–214 (2019).

19. V. Bangari, B. A. Marquez, H. Miller, A. N. Tait, M. A. Nahmias, T. F. de Lima, H.-T. Peng, P. R. Prucnal, B. J. Shastri, Digital electronics and analog photonics for convolutional neural networks (DEAP-CNNs). *IEEE J. Sel. Top. Quantum Electron.* **26**, 1–13 (2020).

20. X. Xu, M. Tan, B. Corcoran, J. Wu, A. Boes, T. G. Nguyen, S. T. Chu, B. E. Little, D. G. Hicks, R. Morandotti, A. Mitchell, D. J. Moss, 11 tops photonic convolutional accelerator for optical neural networks. *Nature* **589**, 44–51 (2021).

21. J. Feldmann, N. Youngblood, M. Karpov, H. Gehring, X. Li, M. Stappers, M. Le Gallo, X. Fu, A. Lukashchuk, A. S. Raja, J. Liu, C. D. Wright, A. Sebastian, T. J. Kippenberg, W. H. P. Pernice, H. Bhaskaran, Parallel convolutional processing using an integrated photonic tensor core. *Nature* **589**, 52–58 (2021).

22. A. Sludds, S. Bandyopadhyay, Z. Chen, Z. Zhong, J. Cochrane, L. Bernstein, D. Bunandar, P. B. Dixon, S. A. Hamilton, M. Streshinsky, A. Novack, T. Baehr-Jones, M. Hochberg, M. Ghobadi, R. Hamerly, D. Englund, Delocalized photonic deep learning on the internet's edge. *Science* **378**, 270–276 (2022).

23. R. Hamerly, L. Bernstein, A. Sludds, M. Soljačić, D. Englund, Large-scale optical neural networks based on photoelectric multiplication. *Phys. Rev. X* **9**, 021032 (2019).

24. T. Wang, S.-Y. Ma, L. G. Wright, T. Onodera, B. C. Richard, P. L. McMahon, An optical neural network using less than 1 photon per multiplication. *Nat. Commun.* **13**, 123 (2022).

25. N. H. Farhat, D. Psaltis, A. Prata, E. Paek, Optical implementation of the hopfield model. *Appl. Optics* **24**, 1469 (1985).

26. S. Kung, H. Liu, "An optical inner-product array processor for associative retrieval," in *Nonlinear Optics and Applications*, Vol. 613 (International Society for Optics and Photonics, 1986), pp. 214–219.

27. J. Ohta, M. Takahashi, Y. Nitta, S. Tai, K. Mitsunaga, K. Kjuma, “A new approach to a gaas/algaas optical neurochip with three layered structure,” in *Proceeding of the IJCNN International Joint Conference on Neural Networks*, Vol. 2 (IEEE, 1989), pp. 477–482.

28. Y. Zuo, B. Li, Y. Zhao, Y. Jiang, Y.-C. Chen, P. Chen, G.-B. Jo, J. Liu, S. Du, All-optical neural network with nonlinear activation functions. *Optica* **6**, 1132–1137 (2019).

29. L. Bernstein, A. Sludds, C. Panuski, S. Trajtenberg-Mills, R. Hamerly, D. Englund, Single-shot optical neural network, arXiv:2205.09103 [cs.ET] (2022).

30. E. Khoram, A. Chen, D. Liu, L. Ying, Q. Wang, M. Yuan, Z. Yu, Nanophotonic media for artificial neural inference. *Photonics Res.* **7**, 823–827 (2019).

31. F. Ashtiani, A. J. Geers, F. Aflatouni, An on-chip photonic deep neural network for image classification. *Nature* **1**, 501–506 (2022).

32. Z. Chen, A. Sludds, R. Davis III, I. Christen, L. Bernstein, L. Ateshian, T. Heuser, N. Heermeier, J. A. Lott, S. Reitzenstein, R. Hamerly, D. Englund, Deep learning with coherent VCSEL neural networks. *Nat. Photon.* **17**, 723–730 (2023).

33. H. H. Zhu, J. Zou, H. Zhang, Y. Z. Shi, S. B. Luo, N. Wang, H. Cai, L. X. Wan, B. Wang, X. D. Jiang, J. Thompson, X. S. Luo, X. H. Zhou, L. M. Xiao, W. Huang, L. Patrick, M. Gu, L. C. Kwek, A. Q. Liu, Space-efficient optical computing with an integrated chip diffractive neural network. *Nat. Commun.* **13**, 1044 (2022).

34. W. Liu, T. Fu, Y. Huang, R. Sun, S. Yang, H. Chen, C-DONN: Compact diffractive optical neural network with deep learning regression. *Opt. Express* **31**, 22127–22143 (2023).

35. X. Lin, Y. Rivenson, N. T. Yardimci, M. Veli, Y. Luo, M. Jarrahi, A. Ozcan, All-optical machine learning using diffractive deep neural networks. *Science* **361**, 1004–1008 (2018).

36. X. Luo, Y. Hu, X. Ou, X. Li, J. Lai, N. Liu, X. Cheng, A. Pan, H. Duan, Metasurface-enabled on-chip multiplexed diffractive neural networks in the visible. *Light Sci. Appl.* **11**, 158 (2022).

37. A. N. Tait, T. F. De Lima, M. A. Nahmias, H. B. Miller, H.-T. Peng, B. J. Shastri, P. R. Prucnal, Silicon photonic modulator neuron. *Phys. Rev. Appl.* **11**, 064043 (2019).

38. J. K. George, A. Mehrabian, R. Amin, J. Meng, T. F. De Lima, A. N. Tait, B. J. Shastri, T. El-Ghazawi, P. R. Prucnal, V. J. Sorger, Neuromorphic photonics with electro-absorption modulators. *Opt. Express* **27**, 5181–5191 (2019).

39. I. A. Williamson, T. W. Hughes, M. Minkov, B. Bartlett, S. Pai, S. Fan, Reprogrammable electro-optic nonlinear activation functions for optical neural networks. *IEEE J. Sel. Top. Quantum Electron.* **26**, 1–12 (2019).

40. A. Jha, C. Huang, P. R. Prucnal, Reconfigurable all-optical nonlinear activation functions for neuromorphic photonics. *Opt. Lett.* **45**, 4819–4822 (2020).

41. C. Huang, A. Jha, T. F. De Lima, A. N. Tait, B. J. Shastri, P. R. Prucnal, On-chip programmable nonlinear optical signal processor and its applications. *IEEE J. Sel. Top. Quantum Electron.* **27**, 1–11 (2021).

42. J. Crnjanski, M. Krstić, A. Totović, N. Pleros, D. Gvozdić, Adaptive sigmoid-like and prelu activation functions for all-optical perceptron. *Opt. Lett.* **46**, 2003–2006 (2021).

43. J. R. Basani, M. Heuck, D. R. Englund, S. Krastanov, All-photonic artificial neural network processor via non-linear optics, arXiv:2205.08608 [physics.optics] (2022).

44. L. G. Wright, T. Onodera, M. M. Stein, T. Wang, D. T. Schachter, Z. Hu, P. L. McMahon, Deep physical neural networks trained with backpropagation. *Nature* **601**, 549–555 (2022).

45. M. Streshinsky, A. Novack, R. Ding, Y. Liu, A. E.-J. Lim, P. G.-Q. Lo, T. Baehr-Jones, M. Hochberg, Silicon parallel single mode 48× 50 gb/s modulator and photodetector array. *J. Lightwave Technol.* **32**, 4370–4377 (2014).

46. S. Kumar, V. Bitorff, D. Chen, C. Chou, B. Hechtman, H. J. Lee, N. Kumar, P. Mattson, S. Wang, T. Wang, Y. Xu, Z. Zhou, Scale mlperf-0.6 models on google tpu-v3 pods, arXiv:1909.09756 [cs.LG] (2019).

47. S. Chen, Y.-C. Liang, S. Sun, S. Kang, W. Cheng, M. Peng, Vision, requirements, and technology trend of 6g: How to tackle the challenges of system coverage, capacity, user data-rate and movement speed. *IEEE Wirel Commun* **27**, 218–228 (2020).

48. M. I. Maulana, M. Suryanegara, "Progress in 6G technology: A short review," in *2023 6th International Conference of Computer and Informatics Engineering (IC2IE)* (IEEE, 2023),pp. 36–41.

49. M. Secondini, E. Forestieri, Scope and limitations of the nonlinear shannon limit. *J. Lightwave Technol.* **35**, 893–902 (2017).

50. S. A. Derevyanko, J. E. Prilepsky, S. K. Turitsyn, Capacity estimates for optical transmission based on the non-linear fourier transform. *Nat. Commun.* **7**, 12710 (2016).

51. R.-J. Essiambre, G. Kramer, P. J. Winzer, G. J. Foschini, B. Goebel, Capacity limits of optical fiber networks. *J. Lightwave Technol.* **28**, 662–701 (2010).

52. A. D. Ellis, J. Zhao, D. Cotter, Approaching the non-linear shannon limit. *J. Lightwave Technol.* **28**, 423–433 (2010).

53. G. Caruso, I. N. Cano, D. Nesset, G. Talli, R. Gaudino, Real-time 100 Gb/s PAM-4 for access links with up to 34 dB power budget. *J. Lightwave Technol.* **41**, 3491–3497 (2023).

54. X. Pang, T. Salgals, H. Louchet, D. Che, M. Gruen, Y. Matsui, T. Dippon, R. Schatz, M. Joharifar, B. Krüger, F. Pittala, Y. Fan, A. Udalcovs, L. Zhang, X. Yu, S. Spolitis, V. Bobrovs, S. Popov, O. Ozolins, 200 Gb/s optical amplifier-free IM/DD transmissions using a directly modulated O-band DFB+R laser targeting LR applications. *J. Lightwave Technol.* **41**, 3635–3641 (2023).

55. P. Torres-Ferrera, G. Rizzelli, H. Wang, V. Ferrero, R. Gaudino, Experimental demonstration of 100 Gbps/λ C-band direct-detection downstream PON using non-linear and CD compensation with 29 dB+ OPL over 0 km–100 km. *J. Lightwave Technol.* **40**, 547–556 (2022).

56. G. Mourgias-Alexandris, A. Tsakyridis, N. Passalis, M. Kirtas, A. Tefas, T. Rutirawut,F. Y. Gardes, N. Pleros, M. Moralis-Pegios, "25GMAC/sec/axon photonic neural networks with 7GHz bandwidth optics through channel response-aware training," in *2021 European Conference on Optical Communication (ECOC)* (IEEE, 2021), pp. 1–4.

57. M. Moralis-Pegios, G. Mourgias-Alexandris, A. Tsakyridis, G. Giamougiannis, A. Totovic, G. Dabos, N. Passalis, M. Kirtas, T. Rutirawut, F. Y. Gardes, A. Tefas, N. Pleros, Neuromorphic silicon photonics and hardware-aware deep learning for high-speed inference. *J. Lightwave Technol.* **40**, 3243–3254 (2022).

58. MathWorks. Modulation classification with deep learning. https://www.mathworks.com/help/comm/ug/modulation-classification-with-deep-learning.html.

59. D. M. B. Lesko, H. Timmers, S. Xing, A. Kowligy, A. J. Lind, S. A. Diddams, A six-octave optical frequency comb from a scalable few-cycle erbium fibre laser. *Nat. Photonics* **15**, 281–286 (2021).

60. F. Couny, F. Benabid, P. Roberts, P. Light, M. Raymer, Generation and photonic guidance of multi-octave optical-frequency combs. *Science* **318**, 1118–1121 (2007).

61. V. J. Urick, K. J. Williams, J. D. McKinney, *Fundamentals of microwave photonics* (John Wiley & Sons, 2015).

62. M. Hochberg, T. Baehr-Jones, G. Wang, J. Huang, P. Sullivan, L. Dalton, A. Scherer, Towards a millivolt optical modulator with nano-slot waveguides. *Opt. Express* **15**, 8401–8410 (2007).

63. D. L. Bhargav, D. Achish, G. Yashwanth, N. P. Kalyan, K. Ashesh, "Prediction of signal drop due to rain at user cellular signal reception," in *Sentimental Analysis and Deep Learning* (Springer, 2022), pp. 357–367.

64. L. Chrostowski, M. Hochberg, *Silicon Photonics Design: From Devices to Systems* (Cambridge Univ. Press, 2015).
